# The Mammalian Orthologs of *Drosophila* Lgd, CC2D1A and CC2D1B, Function in the Endocytic Pathway, but Their Individual Loss of Function Does Not Affect Notch Signalling

**DOI:** 10.1371/journal.pgen.1005749

**Published:** 2015-12-31

**Authors:** Nadja Drusenheimer, Bernhard Migdal, Sandra Jäckel, Lena Tveriakhina, Kristina Scheider, Katharina Schulz, Jieny Gröper, Karl Köhrer, Thomas Klein

**Affiliations:** 1 Institut für Genetik, Heinrich-Heine-Universität Düsseldorf, Düsseldorf, Germany; 2 German Center for Neurodegenerative Diseases (DZNE), Tübingen, Germany; 3 Institut für Molekularbiologie OE5250, Medizinische Hochschule Hannover, Hannover, Germany; 4 Biological and Medical Research Center (BMFZ), Genomics and Transcriptomics Laboratory (GTL), Heinrich-Heine-Universität Düsseldorf, Düsseldorf, Germany; University of Cambridge, UNITED KINGDOM

## Abstract

CC2D1A and CC2D1B belong to the evolutionary conserved Lgd protein family with members in all multi-cellular animals. Several functions such as centrosomal cleavage, involvement in signalling pathways, immune response and synapse maturation have been described for CC2D1A. Moreover, the *Drosophila melanogaster* ortholog Lgd was shown to be involved in the endosomal trafficking of the Notch receptor and other transmembrane receptors and physically interacts with the ESCRT-III component Shrub/CHMP4. To determine if this function is conserved in mammals we generated and characterized *Cc2d1a* and *Cc2d1b* conditional knockout mice. While *Cc2d1b* deficient mice displayed no obvious phenotype, we found that *Cc2d1a* deficient mice as well as conditional mutants that lack CC2D1A only in the nervous system die shortly after birth due to respiratory distress. This finding confirms the suspicion that the breathing defect is caused by the central nervous system. However, an involvement in centrosomal function could not be confirmed in *Cc2d1a* deficient MEF cells. To analyse an influence on Notch signalling, we generated intestine specific *Cc2d1a* mutant mice. These mice did not display any alterations in goblet cell number, proliferating cell number or expression of the Notch reporter Hes1-emGFP, suggesting that CC2D1A is not required for Notch signalling. However, our EM analysis revealed that the average size of endosomes of *Cc2d1a* mutant cells, but not *Cc2d1b* mutant cells, is increased, indicating a defect in endosomal morphogenesis. We could show that CC2D1A and its interaction partner CHMP4B are localised on endosomes in MEF cells, when the activity of the endosomal protein VPS4 is reduced. This indicates that CC2D1A cycles between the cytosol and the endosomal membrane. Additionally, in rescue experiments in *D*. *melanogaster*, CC2D1A and CC2D1B were able to functionally replace Lgd. Altogether our data suggest a functional conservation of the Lgd protein family in the ESCRT-III mediated process in metazoans.

## Author Summary

The proteins of the Lgd/CC2D1 family are conserved in all multicellular animals. The *Drosophila melanogaster* ortholog Lgd is involved in the regulation of signalling receptor degradation via the endosomal pathway. Loss of *lgd* function causes ectopic ligand-independent activation of the Notch signalling pathway due to a defect in the endosomal pathway. For the mammalian proteins no endosomal function has been defined so far. Here, we asked whether the function of Lgd is conserved in mammals with the focus on the question whether its orthologs are also involved in the endosomal pathway and regulation of Notch pathway activity. Therefore, we generated and characterised *Cc2d1a* and *Cc2d1b* conditional knockout mice. We found that the loss of *Cc2d1b* does not lead to an obvious phenotype, while the known lethality of *Cc2d1a* deficient newborns is nervous system dependent. In experiments with MEFs isolated from knockout animals we provide evidence that both CC2D1 proteins are involved in the function of the ESCRT-III complex in a similar manner as Lgd in *D*. *melanogaster*. Moreover, we found that the loss of one CC2D1 protein is not sufficient to cause ectopic activation of Notch signalling.

## Introduction

CC2D1A (Coiled-coil and C2 domain-containing protein 1A)/LGD2 and CC2D1B/LGD1 belong to the evolutionary conserved Lgd protein family that is present in the genomes of all metazoans [1]. Members of this family contain four tandem repeats of the DM14 domain and one C2 domain. The *D*. *melanogaster* ortholog Lgd (Lethal (2) giant discs) was shown to be involved in the trafficking of the Notch receptor and other transmembrane proteins through the endocytic pathway. Loss of its function results in an ectopic and ligand-independent activation of the Notch pathway in several tissues that leads to over-proliferation of imaginal disc cells [1–4]. Therefore *lgd* was classified as a hyperplastic tumour suppressor gene [5]. Moreover, its loss causes enhanced activation of the *D*. *melanogaster* BMP signalling pathway (Dpp pathway) during oogenesis [6].

Several rather diverse functions have been described for CC2D1A. It was first identified in a large-scale screen to identify genes that activate the NFκB pathway in HEK293 cells [7]. Later, the function in the canonical IKK pathway was confirmed [8]. Additionally, CC2D1A appears to act as a transcriptional repressor of the dopamine receptor gene *DRD2* [9] and the serotonin receptor gene *5-HT1A* [10]. Likewise, CC2D1B functions as a repressor for the *5-HT1A* gene [11]. Moreover, CC2D1A seems to be involved in the regulation of signalling pathways. During EGFR signalling it acts as a scaffold protein to recruit and activate PDK1/Akt [12]. In line with this observation is that silencing of *CC2D1A* inhibits growth of EGFR induced lung cancer cells [13]. It is a positive regulator of the cAMP/PKA pathway, where it is required for PKA activation and regulation of PDE4D [14,15]. In innate immunity, CC2D1A modulates the TLR3 and TLR4 signalling pathways [16] and the RLR pathways [17]. Centrosome associated CC2D1A appears to regulate centriole cohesion by preventing premature cleavage in HeLa cells [18]. However, most of these ascribed diverse functions are based on cell culture experiments and it is not known whether the CC2D1 proteins perform them *in vivo*.

Several mutations in *CC2D1A* are linked to severe forms of intellectual disability and autism spectrum disorder in humans [19,20]. *Cc2d1a* deficiency in mice leads to an even severer phenotype [15,17,21]. Mutant mice die postnatally within a few minutes [21], hours [15] or within a day after birth [17] due to difficulties in breathing. The underlying cause of the defect was suspected to be a defect in maturation of synapses [21] or dysregulation of the cAMP/PKA pathway [15]. However, the analysis was performed with conventional knockout mice and it remains unclear which mutant tissue contributes to the breathing defect. In contrast to CC2D1A, virtually nothing is known about the function of CC2D1B *in vivo*.

In *D*. *melanogaster*, Lgd physically interacts with Shrub (CHMP4 in mammals), the major subunit of the ESCRT-III complex, which is required for the formation of intraluminal vesicles (ILVs) during endosomal maturation [22]. ESCRT-III together with four other ESCRT-complexes generates the ILVs in maturing endosomes (MEs), which—as a result—become multivesicular bodies (MVBs). In *D*. *melanogaster*, ILV formation is required for the termination of signalling through several signalling pathways, among them the BMP and EGFR-pathways. Moreover, it prevents the ectopic activation of the Notch pathway [23–25]. Whether this is also the case in mammals is not known. The other ESCRT-complexes are termed ESCRT-0, I, II, and the VPS4 complex. ESCRT-0—II act in sequence to concentrate ubiquitinated cargo and to assemble ESCRT-III at the sites of cargo concentration. ESCRT-III consists of four subunits (CHMP6, CHMP4, CHMP3 and CHMP2) as well as several auxiliary factors (reviewed in [26]). Three *CHMP4* genes are present in humans, termed *CHMP4A*, *B* and *C*. Mice lack *CHMP4A* [27]. CHMP6 is activated by ESCRT-II and induces the polymerisation of CHMP4s into a filament that is capped by CHMP3 and CHMP2. The filament is disassembled by the AAA ATPase VPS4 into monomers during vesicle abscission. CHMP4 monomers exist in a “closed” inactive form in the cytosol and can be recruited to the limiting membrane for another round of polymerisation. Thus, CHMP4s cycle between a monomeric closed form in the cytosol and an “open” polymerised form at the endosomal membrane. The cycling is the reason for the finding that the majority of CHMP4s are located in the cytosol upon antibody staining. Only if the function of Vps4p is abolished, ESCRT-III proteins strongly accumulate at the endosome and can be detected there in antibody staining [28].

Although clearly involved in endosomal trafficking in *D*. *melanogaster*, no definite link to endocytic trafficking has been established for the mammalian orthologs of Lgd, CC2D1A and CC2D1B. Rescue experiments in *D*. *melanogaster* suggested functional similarities, since murine CC2D1A and CC2D1B were able to replace Lgd during wing development [1]. However, it turned out later that the rescue assay was not suitable to test the ability of the Lgd orthologs to functionally replace Lgd [22]. Another assays suggested that down-regulation of *CC2D1A* in HeLa cells weakly decreased EGF and TF endocytosis [29]. A yeast two-hybrid screen revealed an interaction between CC2D1A and CHMP4 proteins [30]. This interaction was further refined by Martinelli et al. by showing that CC2D1A might regulate CHMP4B polymerisation [31]. The same group could also show that the over-expression of CC2D1A inhibits CHMP4B dependent HIV-1-budding in cells. Correspondingly, siRNA mediated depletion of *CC2D1A* increased HIV-1-budding under certain conditions [32]. Taken together, these experiments suggest that mammalian members of the Lgd gene family might also participate in the regulation of the endocytic pathway.

Like in *D*. *melanogaster*, the Notch signalling pathway plays a pivotal role during development and tissue homeostasis in mammals (reviewed in [33]). In the gut epithelium, the role of the Notch pathway is particularly well understood. It is required for the maintenance of proliferation of intestinal stem cells and for the differentiation of the progenitor cells in the crypt. Inhibition of Notch signalling results in the arrest of crypt cell proliferation and guides all crypt cells into a goblet cell fate [34]. Conversely, ectopic Notch signalling in the embryonic and adult intestine leads to ectopic proliferation of crypt progenitor cells and impairs goblet and enteroendocrine cell differentiation [35,36].

The Notch signalling pathway is activated through direct binding of its ligands (reviewed in [37]). The binding elicits the separation of the intracellular domain of the receptor (NICD) through two proteolytic cleavages by ADAM10 and the γ-secretase complex, which subsequently travels to the nucleus and associates with the CSL transcription factor to activate expression of target genes, such as *Hes1*.

Here, we analyse the role of CC2D1A and CC2D1B in mouse with the focus on the questions whether CC2D1 proteins are involved in the endocytic pathway and the regulation of the activity of Notch signalling as observed for Lgd in *D*. *melanogaster*. For this purpose, we have generated conditional knockout mouse models for both genes and analysed their function. We show for the first time that the postnatal death of *Cc2d1a* deficient mice is caused by tissue-specific absence of the gene function in the nervous system. In contrast, *Cc2d1b* does not appear to be essential for embryogenesis or survival or fertility of mice. Furthermore, we find that Notch signalling is not altered in the intestine of *Cc2d1a* or *Cc2d1b* deficient mice compared to wild type. Murine embryonic fibroblasts (MEFs) derived from mutant animals do not show detectable alterations in the endocytic trafficking and degradation of the Notch receptor. We observe that CC2D1A, like *D*. *melanogaster* Lgd, locates in the cytosol, but accumulates at MEs when VPS4 function is reduced. This finding, together with cell fractionation data, indicates that CC2D1 proteins are temporally associated with the ME. EM analysis of MEFs reveals an increase of the size of the endosome in *Cc2d1a* deficient cells, indicating a defect in endosomal morphogenesis. In *D*. *melanogaster*, expression of CC2D1A and CC2D1B under the control of the endogenous *lgd* promoter can rescue the loss of function of *lgd*. Altogether, the results provide strong evidence that the endosomal function of the Lgd protein is conserved in mammals.

## Results

### *Cc2d1b* is not essential during embryogenesis or survival and fertility of adult mice

**Fig 1:**
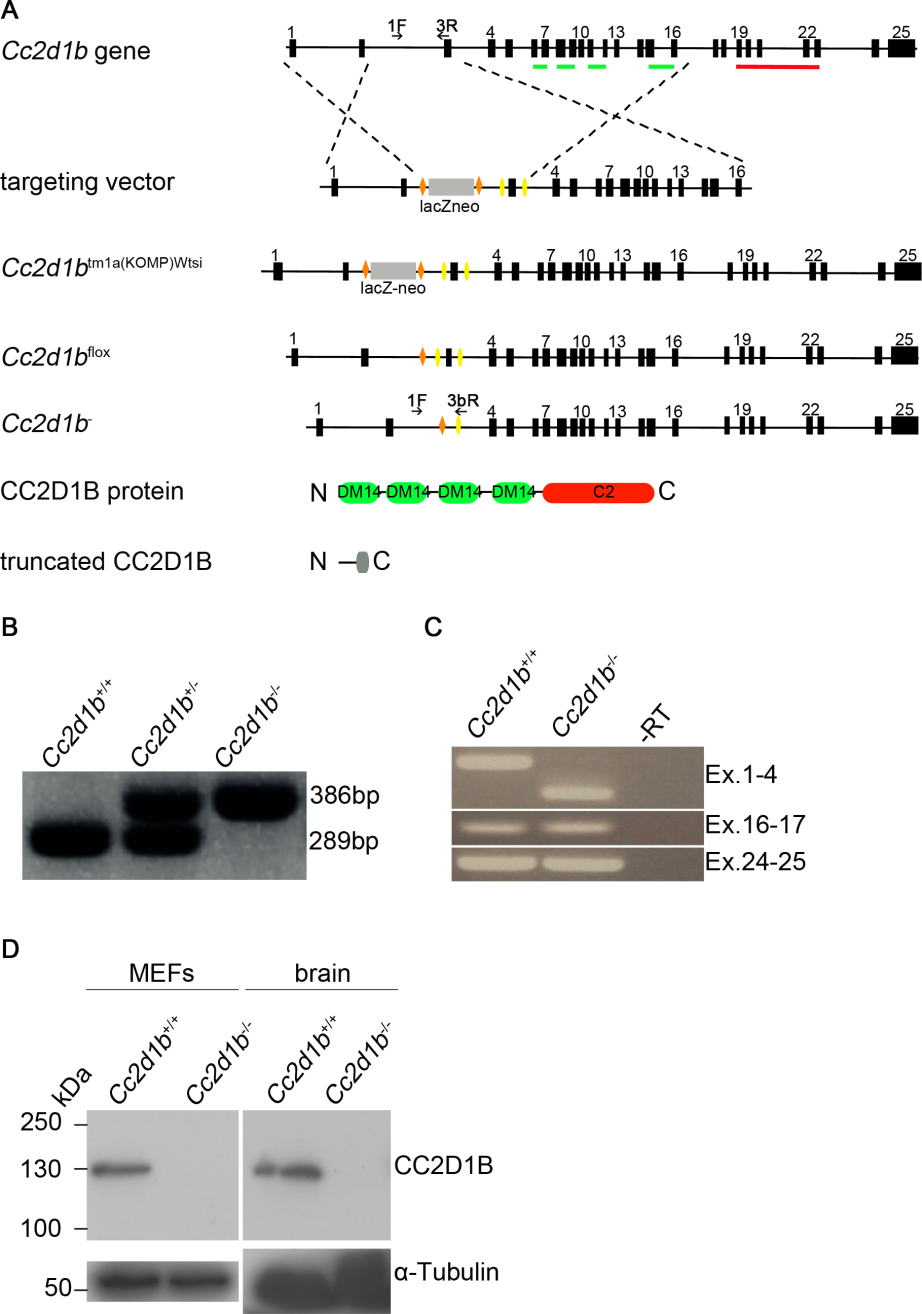
Generation of a conditional knockout mouse of the *Cc2d1b* gene locus. (A) Scheme for generating animals carrying the conditional knockout allele (*Cc2d1b* ^flox^) and the recombined allele (*Cc2d1b* ^*-*^). LoxP sites are depicted as yellow triangles, FRT sites as orange triangles. The DM14 and C2 protein domains are marked in green and red, respectively. Primers for genotyping are depicted as black arrows. (B) PCR genotyping of wild-type and heterozygous and homozygous *Cc2d1b* ^*-*^ mice using primers 1F, 3R and 3bR. (C) RT-PCR analysis of total RNA extracts of brain of newborn animals with exon specific primer pairs that confirm lack of expression of the deleted segment. (D) Immunoblotting of protein lysates from MEF (mouse embryonic fibroblast) cells isolated from *Cc2d1b* ^-/-^ and control mice. The 130kDa CC2D1B band is lacking in the lysates of homozygous *Cc2d1b* ^-/-^ mice.

To characterise the function of CC2D1B *in vivo* we generated a *Cc2d1b* conditional null allele by breeding the KOMP mouse strain C57BL/6N-*Cc2d1b* ^tm1a(KOMP)Wtsi^ with a Flp deleter strain to generate a *Cc2d1b* allele where exon 3 is flanked by loxP sites (Fig 1A). Cre-mediated recombination deletes the floxed exon, leading to a frame-shift mutation and a premature stop codon after 102bp of missense sequence. The predicted truncated protein consists of 56 amino acids and contains no known domains. We confirmed *Cc2d1b* deficiency by genotyping PCR, RT-PCR and immunoblotting (Fig 1B–1D). For immunoblotting, we generated a polyclonal antibody directed against the N-Terminus of CC2D1B (aa 1–253). The truncated protein was not detectable, suggesting low affinity of the polyclonal antibody to epitopes in the truncated protein, nonsense-mediated mRNA decay or instability of the truncated CC2D1B protein. Nevertheless, a putative dominant negative effect of such a fragment is highly unlikely as the fragment lacks all known functional domains. The genotype distribution of offspring from heterozygous breedings matched the expected Mendelian ratio (n = 30; wild type = 6, heterozygous = 17, homozygous = 7; χ^2^ = 0.59, p = 0.74). Homozygous animals are viable and fertile with no obvious abnormalities and were kept as a homozygous line. In summary, loss of *Cc2d1b* function does not lead to an obvious phenotype under the used conditions.

One possibility why the loss of *Cc2d1b* function has no detectable effect on viability of mice is that the expression of CC2D1A is up-regulated to compensate for its loss. We tested this possibility in Western blots and found that the expression of CCD1A was unaffected in *Cc2d1b* deficient MEFs, instead of elevated as would be expected (S1A and S1C Fig). Conversely, the loss of *Cc2d1a* caused a reduction of the CC2D1B level to approximately 60% of the wild type level (S1A and S1B Fig; see below for *Cc2d1a* mutant cells). Thus, the lack of a mutant phenotype of *Cc2d1b* mutants cannot be explained by a compensatory up-regulation of the expression of CC2D1A.

### *Cc2d1a* deficient mice die shortly after birth due to respiratory distress

**Fig 2:**
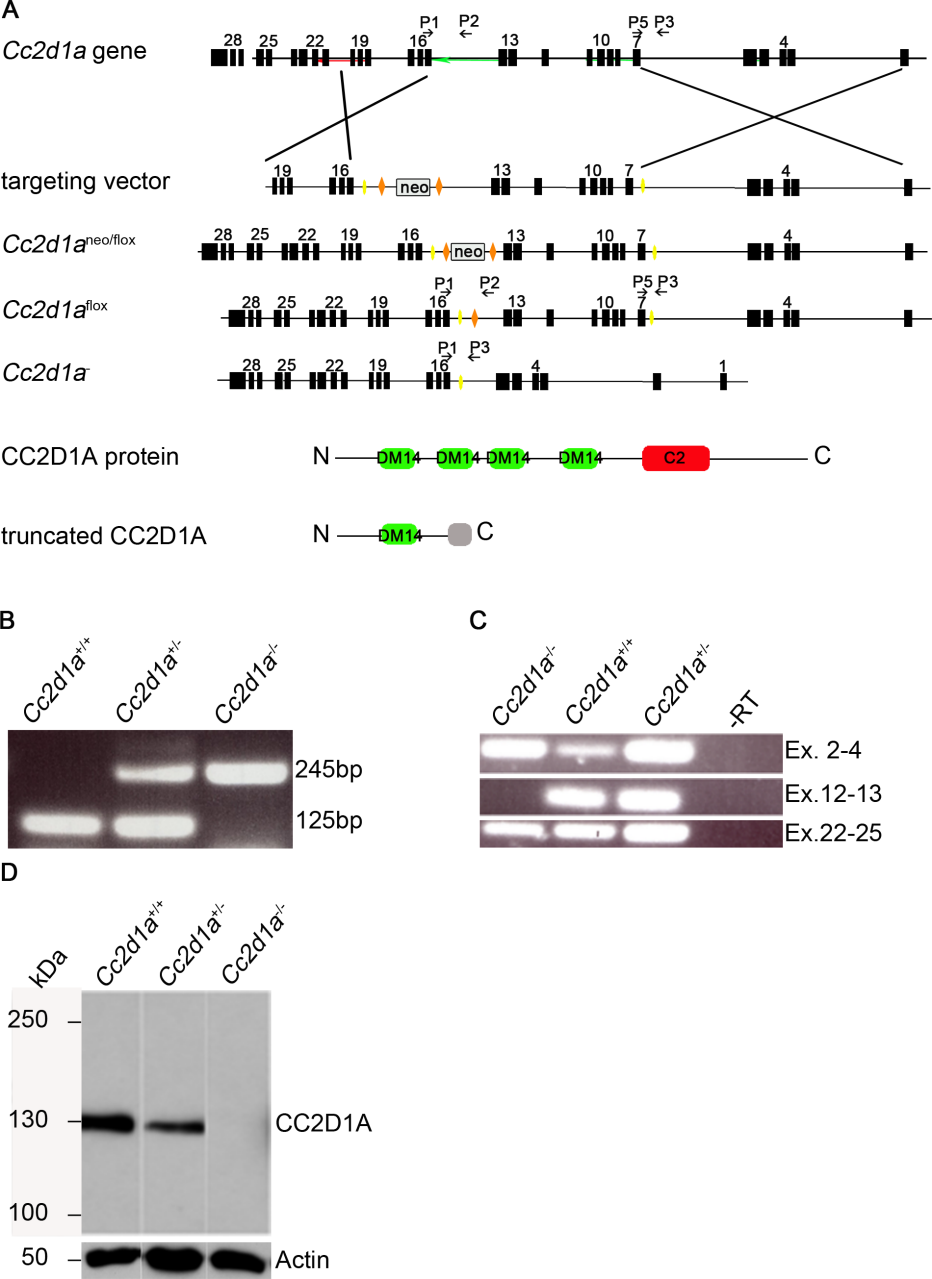
Generation of a conditional knockout mouse of the *Cc2d1a* gene locus. (A) Scheme for generating animals carrying the conditional knockout allele (*Cc2d1a* ^flox^) and the recombined allele (*Cc2d1a* ^-^). LoxP sites are depicted as yellow triangles, FRT sites as orange triangles. The DM14 and C2 protein domains are marked in green and red, respectively. Primers for genotyping are numbered P1-P5 and are depicted as black arrows. (B) PCR genotyping of wild type and heterozygous and homozygous *Cc2d1a* ^-^ mice using primers P1, P3 and P5. (C) RT-PCR analysis of total RNA extracts of brain of newborn animals with exon specific primer pairs that confirm lack of expression of the deleted segment. (D) Immunoblotting of protein lysates from MEF cells isolated from *Cc2d1a* ^*-/-*^ and control mice. The 130kDa CC2D1A band is lacking in the lysates of homozygous *Cc2d1a* ^-/-^ mice.

To characterise the function of CC2D1A *in vivo* we generated a *Cc2d1a* conditional null allele where the exons 7 and 14 are flanked by loxP sites (Fig 2A). Cre-mediated recombination leads to deletion of the floxed DNA segment. In the resulting *Cc2d1a* transcript exon 6 is spliced to exon 15, leading to a frame-shift mutation and a premature stop codon after 120bp of missense sequence. The truncated protein consists of 284 amino acids and contains just the first DM14 domain (Fig 2A). Homologous recombination of ES cells was analysed by Southern Blotting and subsequently functionality of loxP sites in ES cell clones was confirmed by a cell permeable His-TAT-NLS-Cre [38] prior to blastocyst injection. *Cc2d1a* ^neoflox^ mice were bred with Flp deleter and Cre deleter lines and *Cc2d1a* deficiency was confirmed by genotyping PCR, RT-PCR and Immunoblotting (Fig 2B–2D). The truncated protein could not be detected.

**Fig 3:**
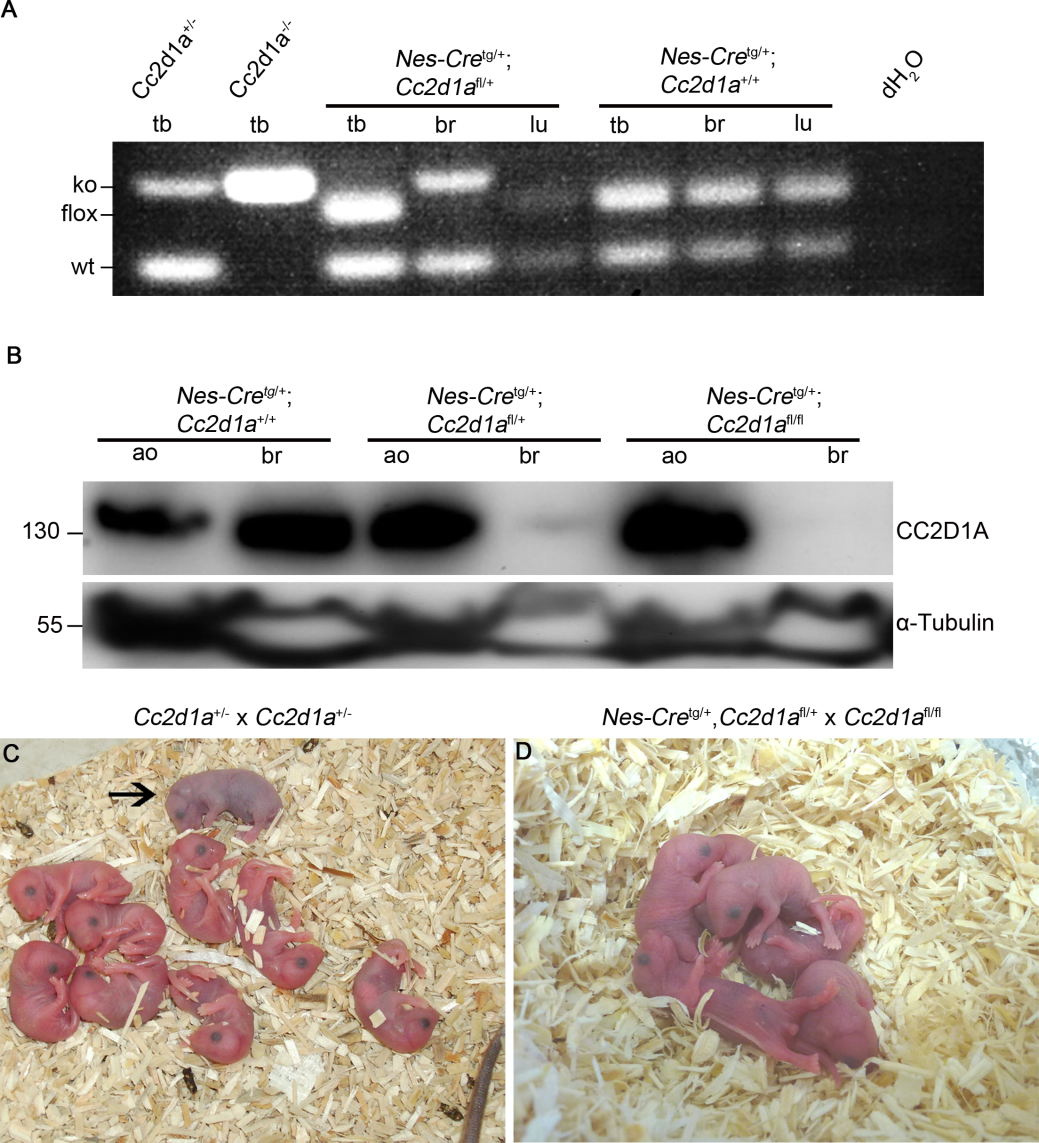
Disruption of *Cc2d1a* in the nervous system results in perinatal death. (A) PCR genotyping of different tissues (tb: tail biopsy, br: brain, lu: lung) of Nestin-Cre mediated wild type and heterozygous *Cc2d1a* mutants using primers P1,P3 and P5. DNA from heterozygous and homozygous *Cc2d1a* deficient mice was used as control. Cre mediated recombination of *Cc2d1a* is only detected in the brain of mutant mice. (B) Immunoblotting of protein lysates from abdominal organs (ao) and brain (br) isolated from *Nestin-Cre* ^tg/+^;*Cc2d1a* ^flox/flox^ and control mice. The 130kDa CC2D1A band is absent in the lysates of brain of homozygous mutant mice but not in lysates of abdominal organs or control siblings. (C, D) Litter of breedings of full knockout animals (C) and of conditional brain specific knockout animals (D). Homozygous mutants from full knockouts could not start breathing and turned cyanotic (marked by an arrow). One third of homozygous conditional mutants developed the same phenotype after delivery, while about two-thirds of homozygous conditional mutants started breathing and were undistinguishable from littermates but died within a day.

Heterozygous animals are viable, fertile and display no obvious abnormalities. Genotyping of litter from heterozygous breedings revealed no homozygous littermates, suggesting that no homozygous animal survive until weaning age (3–4 weeks). Closer examination of newborn pubs revealed that homozygous mice die within a few hours after delivery, similar to recently published studies [15,17,21]. Size and weight of the homozygous pups did not differ from their littermates but they displayed severe difficulties in breathing and turned cyanotic within a few minutes, suggesting that these pups suffer from respiratory distress (see Fig 3C).

### Loss of *Cc2d1a* in the nervous system results in perinatal death

An open question is whether a defect in the brain is responsible for the breathing defect that causes the perinatal death of *Cc2d1a* deficient mice [21]. This assumption could not be proven, as no conditional knockout was available. Our conditional knockout mouse model enabled us to test this hypothesis. To do so, we crossed *Cc2d1a* ^flox/flox^ mice with *Nestin*-*Cre* ^tg/+^ mice, which lead to brain specific inactivation of *Cc2d1a* [39]. Cre-mediated recombination of the *Cc2d1a* ^flox^ allele in CNS neurons and glia cells was confirmed by PCR analysis of DNA extracted from brain and tail biopsies of mutant and wild type embryos (E14.5) and immunoblotting with CC2D1A antibodies (Fig 3A and 3B). Extensive breeding of *Nestin-Cre* ^tg/+^,*Cc2d1a* ^flox/+^ animals with homozygous *Cc2d1a* ^flox/flox^ mice did not lead to any conditional *Cc2d1a* mutant animals at weaning age (S1 Table) suggesting early lethality of the *Nestin-Cre* ^tg/+^,*Cc2d1a* ^flox/flox^ animals. As the genotype distribution of conditional *Cc2d1a* mutant embryos up to E18.5 was normal (S2 Table) we had a closer look at newborn animals and compared *Cc2d1a* deficient animals with Nestin-Cre conditional *Cc2d1a* mutants. We monitored five births for both lines. Mutant animals were born in expected Mendelian ratios (S3 and S4 Tables). Eight out of eight *Cc2d1a* deficient animals failed to breathe and turned cyanotic after birth, while this was the case in only four out of twelve brain specific *Cc2d1a* mutants (Fig 3C and 3D). Nevertheless, all brain specific *Cc2d1a* mutants died within 12 hours after birth, as no animal was observed the morning after giving birth probably due to the mother cannibalizing dead litter.

The slight difference in the time of death between the full knockout animals and the brain specific mutants might suggest that the reason for the perinatal death is not entirely nervous system dependent or that the background of mice influences the time of death. The latter is supported by the fact that *Cc2d1a* deficient mice of a C57BL/6J background [17] die within one day after birth while *Cc2d1a* deficient mice of 129/Sv background die within minutes after birth [21]. As *Cc2d1a* mice in this study were derived from a C57BL/6N x 129Sv hybrid line backcrossed several generations to C57BL/6N and the Nestin-Cre animals are of a C57BL/6J genetic background, the observed difference might be strain-dependent. To test this possibility we crossed *Cc2d1a* ^+/-^ animals with C57BL/6J wild type or C57BL/6J-*Nestin-Cre* ^+/-^ mice to generate *Cc2d1a* mutants with the same background as the Nestin-Cre conditional mutants. We monitored three births with five *Cc2d1a* deficient animals (S5 Table). Five out of five mutant animals displayed no breathing after birth and turned cyanotic like the mutants on the original background, suggesting that the analysed backgrounds do not influence the time of death. Taken together, these results indicate that the loss of CC2D1A in the nervous system is sufficient to induce the full knockout phenotype (namely the perinatal death due to respiratory distress), even though with incomplete penetrance.

### CC2D1A appears not to regulate centrosome function or mitotic events in murine cells

**Fig 4:**
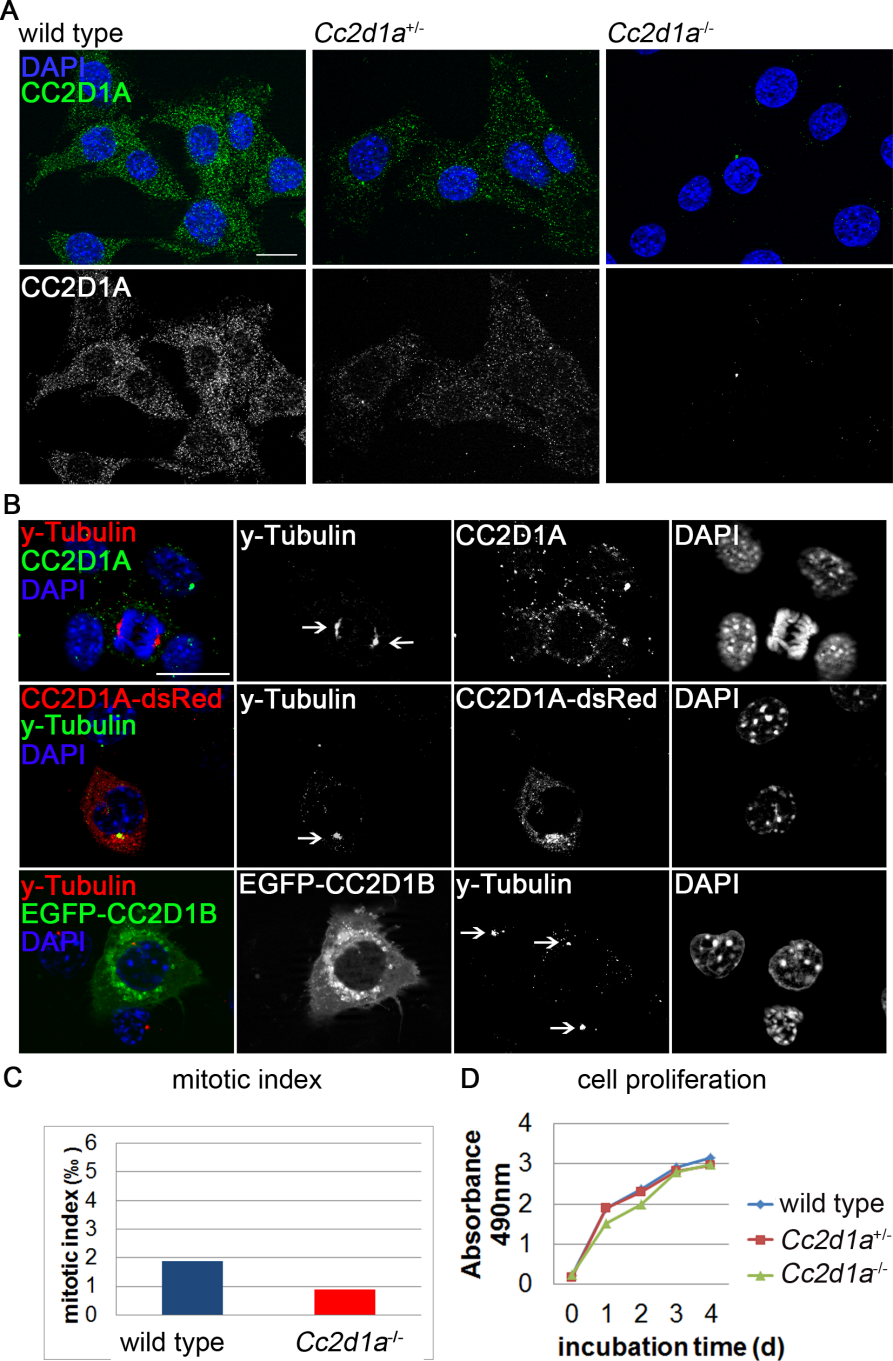
CC2D1A appears not to regulate centrosome function. (A) Immunocytochemical staining of wild type, *Cc2d1a* ^+/-^ and *Cc2d1a* ^-/-^ MEF cells with an affinity purified anti-guinea-pig-CC2D1A antibody. No staining could be detected in *Cc2d1a* deficient cells. (B) Immunocytochemical staining of wild type, CC2D1A-dsRed or EGFP-CC2D1B overexpressing cells revealed 1 or 2 γ-Tubulin positive centrosomes (marked by arrows). CC2D1A and γ-Tubulin co-localise only in over-expression. (C, D) Neither the mitotic index (in 1478 mutant cell and 974 wild type cells) (C) nor cell proliferation (D) is significantly altered in *Cc2d1a* deficient MEF cells compared to wild type MEF cells. Two to three different passages were analysed per genotype. Scale bars are 20 μm.

CC2D1A was recently described to localise to centrosomes and regulate centriole cohesion in HeLa cells. siRNA mediated knockdown of CC2D1A in these cells was shown to cause formation of multipolar spindles accompanied by separase dependent centriole splitting [18]. To review these finding in murine cells we established murine embryonic fibroblast (MEF) cell lines isolated from *Cc2d1a* ^-/-^, *Cc2d1a* ^+/-^ and wild type embryos and stained them with CC2D1A specific antibodies. The commercially available antibodies used in previous studies gave strong signals in our assay in all genotypes suggesting unspecific binding of the antibodies to other cellular antigens (see S2 Fig). To generate a specific polyclonal CC2D1A antibody we immunized guinea pigs with a murine antigen that comprises the 4th DM14 domain (aa 481 to 640) fused to the extended C-Terminus of CC2D1A (aa 788 to 943) that is absent in CC2D1B. Immunocytochemical staining of MEF cells revealed a specific signal of CC2D1A in wild type MEFs, a weaker signal in heterozygous *Cc2d1a* ^+/-^ cells and no signal in homozygous *Cc2d1a* ^-/-^ cells (Fig 4A), confirming the specificity of the antibody.

It is reported that CC2D1A co-localises with the centrosome marker γ-Tubulin in HeLa cells and its depletion causes an increase in the mitotic index and a reduction in the number of viable cells [18]. To confirm this finding in primary murine cells we stained MEF cells with anti-γ-Tubulin and anti-CC2D1A (Fig 4B). We did not find significant co-localisation of γ-Tubulin and CC2D1A. However, when we over-expressed human CC2D1A-dsRed we found co-localisation with γ-Tubulin in several cells (Fig 4B), suggesting that CC2D1A proteins do localise close to the centrosomes upon overexpression. In contrast, human EGFP-CC2D1B does not co-localise with γ-Tubulin upon over-expression (Fig 4B).

We did not observe a significant difference in the mitotic indices of wild type (0.02%, 974 cells) and *Cc2d1a* deficient (0.01%, 1478 cells) MEFs (Fig 4C). Finally, a cell proliferation assay did not reveal significant variation between genotypes (Fig 4D). In summary, while CC2D1A appears to contribute to centrosome function in HeLa cells, we failed to find any evidence for this function in primary murine cells.

### Loss of *Cc2d1a* or *Cc2d1b* in the gut epithelium does not affect Notch signalling

**Fig 5:**
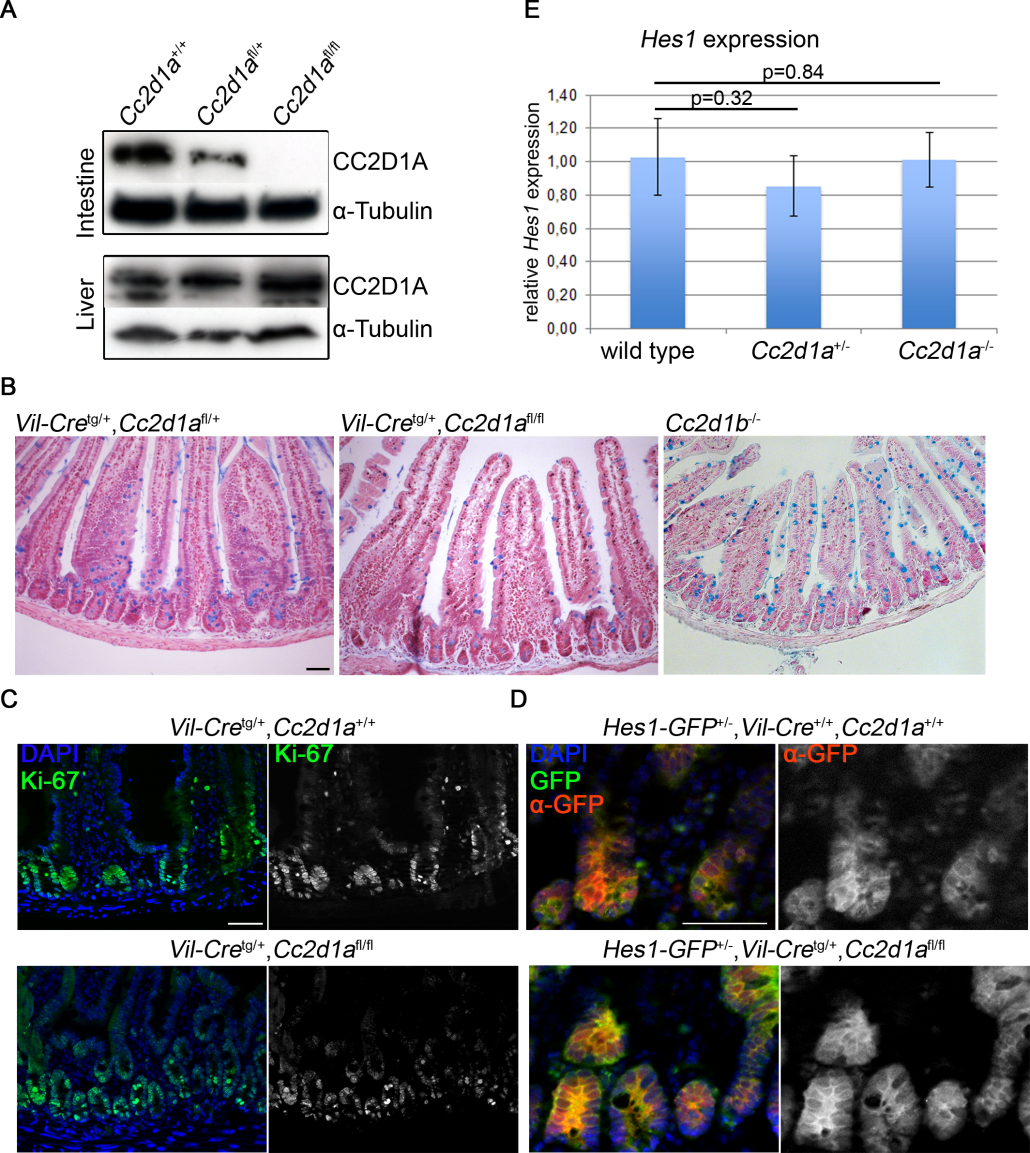
*Cc2d1a* or *Cc2d1b* deficient intestine does not display alterations in goblet cell number, proliferating cells nor Notch signalling. (A) Immunoblotting of protein lysates from liver and intestine isolated from *Villin-Cre* ^tg/+^,*Cc2d1a* ^flox/flox^ and control mice. CC2D1A is expressed in the liver of all analysed animals and in the intestine of heterozygous and wild type mice, but not in homozygous animals. (B) Alcian blue staining of paraffin embedded intestinal sections revealed no alterations in goblet cell number between *Villin-Cre* ^tg/+^,*Cc2d1a* ^flox/flox^, Cc2d1b^-/-^ and wild type animals. (C, D) Immunohistochemical staining was performed on cryosections with antibodies directed against the proliferation marker Ki67 (C) and GFP (D). (E) Relative *Hes1* mRNA levels in intestine of wild type and mutant animals were monitored by qRT-PCR. No differences between mutant and control animals were detected (n = 3). Scale bars are 50 μm.

In *D*. *melanogaster* Lgd was shown to be involved in the regulation of the endocytic trafficking of the Notch receptor and loss of its function results in uncontrolled ectopic activation of the pathway [1,3,4]. To test whether this function in regulation of Notch activity is conserved in mammals, we analysed mice that lack CC2D1A in the intestinal epithelium. The role of Notch signalling during maintenance of this tissue is well understood. To generate the epithelium specific *Cc2d1a* knockout, we bred *Cc2d1a* ^flox/flox^ mice with *Villin-Cre* ^tg/+^ transgenic mice [40]. Loss of CC2D1A was confirmed by Western blotting (Fig 5A). Sections of the small intestine of adult *Villin-Cre* ^*tg/+*^,*Cc2d1a* ^flox/flox^, *Cc2d1b* ^-/-^ and control mice were stained with Nuclear fast red and Alcian blue to label Goblet cells, whose differentiation is dependent on Notch signalling [41]. We found that the overall morphology of the epithelia was similar in wild type and *Cc2d1a* and *Cc2d1b* mutant intestines. Moreover, no significant difference was observed in the number and distribution of goblet cells in the analysed genotypes (Figs 5B and S3). In addition, dividing cells, visualised by staining with the proliferating cell antigen Ki-67, were restricted to the crypts of the small intestine in the *Cc2d1a* mutant and in the wild type (Fig 5C) and no ectopic proliferation of progenitor cells, as seen in animals that ectopically express the constitutively active form of Notch1 (N1ic) [35] could be detected. This indicates that also the Notch dependent proliferation of intestinal progenitor cells is not impaired upon loss of *Cc2d1a* function.

To directly monitor Notch activity in the gut epithelium, we used the recently published Notch reporter line Hes1-EmGFP^SAT^ [42]. In this knock-in line emerald-GFP is expressed under the control of the endogenous promoter of the Notch transcriptional target gene *Hes1*. Comparing GFP signals in cryosections of wild type and Hes1-emGFP intestines revealed high background of fluorescence. To identify putative changes in the GFP expression we immunostained mutant and control intestines with anti-GFP antibodies. GFP was detected mainly in the crypts of the intestine, where Notch signalling is known to regulate stem cell self-renewal and progenitor cells. No obvious differences in the amount of GFP-expressing cells between the analysed genotypes were found (Fig 5D). Furthermore, a quantitative analysis of *Hes1* mRNA expression levels in mutant and control mice revealed no differences between animals (Fig 5E) suggesting that Notch signalling is not altered in mice lacking CC2D1A or CC2D1B in the intestine.

### Endosomal degradation of the NOTCH1 receptor is not altered in *Cc2d1a* deficient MEFs

**Fig 6:**
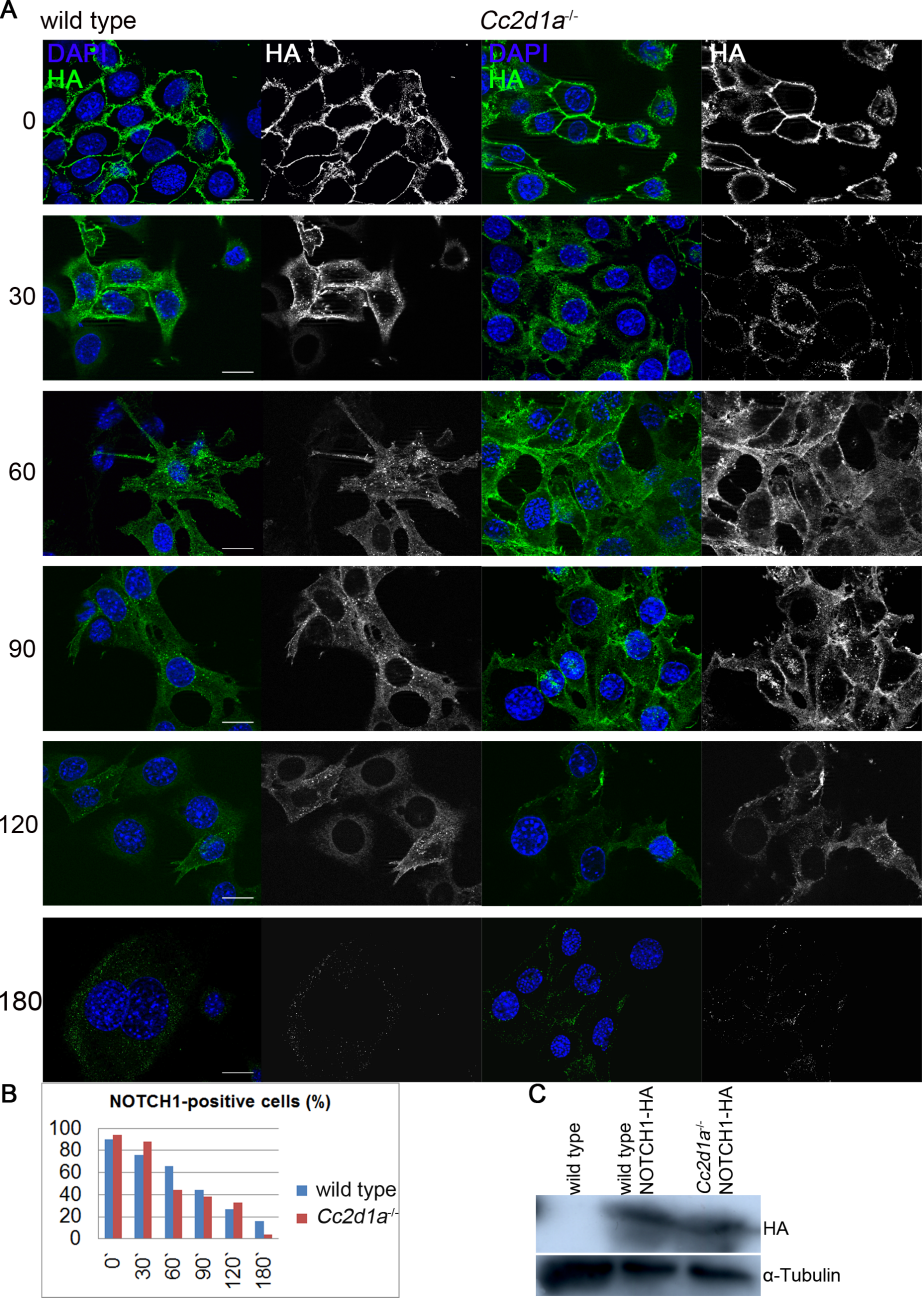
NOTCH1 endocytosis and degradation in wild type and *Cc2d1a* deficient MEFs. (A) Pulse-chase antibody uptake assay. NOTCH1-HA overexpressing MEFs were treated with Alexa 488 coupled anti-HA antibody and endocytosis and degradation was monitored for various points in time. (B) Quantification of NOTCH1 degradation. Stated is the average percentage of cells showing HA staining after the analysed incubation times. A minimum of 50 cells per point in time from at least 2 independent experiments were counted. (C) Immunoblotting of protein lysates from indicated MEF cells. The 300kDa NOTCH1-HA band is detected in transduced cells, but not in wild type cells. Scale bars are 20 μm.

In flies loss of Lgd function causes a general defect in endosomal trafficking that affects several cargo proteins. To analyse whether Notch degradation is impaired in *Cc2d1a* deficient MEF cells, we performed a pulse-chase antibody uptake assay on wild type and *Cc2d1a* deficient MEFs (Fig 6A). For this purpose MEF cells were stably transduced with human NOTCH1 tagged with an HA epitope in its extracellular domain [43]. The generated cell lines display comparable NOTCH1-HA expression levels (Fig 6C). For the assay, they were incubated with an Alexa488-labelled anti-HA antibody at 4°C and then shifted for various periods of time to 37°C (see also [44]). Initially, NOTCH1 was detected at the cell surface and after a 30 min chase NOTCH1 was also visible in intracellular vesicles in both analysed genotypes. After 60 min the receptor was rarely located at the cell surface but in intracellular vesicles. The number of labelled cells decreased constantly, with only very few cells labelled after 180 min of incubation (at 37°C). Calculating the number of fluorescent labelled cells in 2 independent experiments revealed no striking differences between wild type and *Cc2d1a* deficient cells (Fig 6B), suggesting that NOTCH1 endocytosis and degradation is not affected by the lack of CC2D1A.

### CC2D1A and CC2D1B appear to cycle between the cytosol and the limiting membrane of maturing endosomes

To determine the subcellular localisation of CC2D1A, we stained wild type MEFs using our specific CC2D1A antibody together with organelle markers (S4 Fig). We found that CC2D1A is distributed in punctate structures in the cytosol. We could not associate these punctae with distinct organelles, indicating that CC2D1A is not localised on the ER, the Golgi or endosomes but accumulate in unidentified punctae.

**Fig 7:**
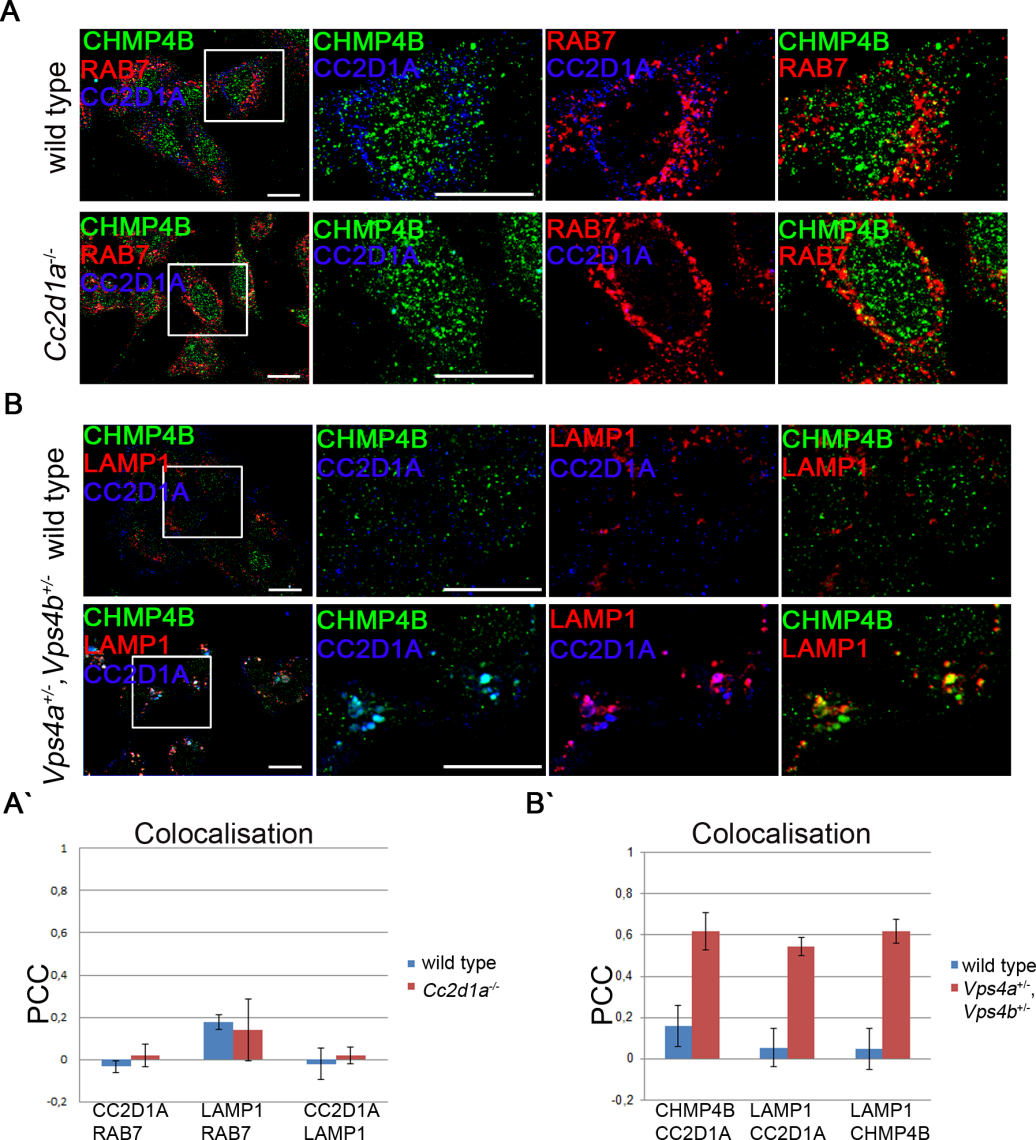
Subcellular localisation of CC2D1A and CHMP4B in wild type cells and *Vps4a* ^+/-^,*Vps4b* ^+/-^ cells. (A) Immunocytochemical staining was performed on wild type and *Cc2d1a* deficient MEFs with antibodies directed against CC2D1A, CHMP4B and the late endosomal marker RAB7. No significant co-localisation of the proteins was detected. Lack of CC2D1A did not alter the distribution of CHMP4B and RAB7. (B) Immunocytochemical staining was performed on wild type and *Vps4a* ^+/-^,*Vps4b* ^+/-^ MEFs with antibodies directed against CC2D1A, CHMP4B and the late endosomal/lysosomal marker LAMP1. In *Vps4a* ^+/-^,*Vps4b* ^+/-^ cells CC2D1A, CHMP4B and LAMP1 co-localise on enlarged vesicles.(A`, B`) Colocalisation was assessed by measuring the Pearson`s correlation coefficient (PCC) (n≥4). Scale bars are 20 μm.

Moreover, we found little co-localisation of CC2D1A and CHMP4B and neither of them could be detected on RAB7 positive late endosomes (Fig 7A and 7A`). CC2D1 proteins have a C2 binding domain that has been shown to be able to bind phospholipids present in endosomal membranes [4]. To test whether CC2D1A and CC2D1B can associate with membranes in general, we performed a cell fractionation assay. We detected CC2D1A and CC2D1B in the cytosolic as well as in the membrane fraction, in contrast to control proteins, which were correctly restricted to only one fraction (S5A Fig). This result indicates that a fraction of CC2D1A and also CC2D1B is associated with membranes. CHMP4 proteins cycle between the endosomal membrane and the cytosol. An endosomal localisation of the CHMP4 yeast ortholog Snf7 could only be observed in the absence of Vps4p function [28]. Loss of Vps4p leads to an accumulation of ESCRT-III on endosomal membranes and disrupts proper ILV formation. We wondered whether this is also the case for CHMP4 proteins in mammals and, since CC2D1 proteins physically interact with CHMP4 proteins (see below), whether CC2D1A also accumulates at the endosomal membrane upon reduction of VPS4 function. The mammalian genome contains two *Vps4* genes, *Vps4a* and *Vps4b* [45]. Homozygous *Vps4a* ^-/-^ and *Vps4b* ^-/-^ mice die during early embryonic stages. To analyse the influence of the two mammalian orthologs VPS4A and VPS4B on subcellular localisation of CC2D1A, we generated MEFs that are double heterozygous for *Vps4a* and *Vps4b*. Strikingly, we observed dramatically enlarged LAMP1 positive MEs/lysosomes in these cells, although the corresponding mice were healthy and displayed no detectable phenotype (Fig 7B). Moreover, CHMP4B accumulated on the enlarged MEs of the double heterozygous cells (Fig 7B and 7B`). This indicates that a reduction of VPS4 function results in the accumulation of CHMP4 proteins on the endosomal membrane also in mammals. Importantly, also CC2D1A accumulated on these CHMP4B/LAMP1 positive vesicles (Fig 7B and 7B`). In a complementary experiment, we over-expressed a dominant negative VPS4B (VPS4B^E235Q^) that lacks its ATPase activity leading to aberrant endosomal structures [46]. Indeed, we could confirm that expression of VPS4B^E235Q^ in wild type MEFs leads to the formation of enlarged endosomal structures while expression of normal VPS4B did not lead to any changes (S5B and S5B` Fig). Moreover, FK2, a marker for ubiquitinated proteins, strongly labelled the VPS4B^E235Q^ induced aberrant MEs, suggesting that the removal of CHMP4 from the endosomal membrane is impaired. As in *Vps4a* ^+/-^,*Vps4b* ^+/-^ cells, CHMP4A and CC2D1A accumulated on the enlarged MEs (S5B and S5B` Fig). These results indicate that CC2D1A might function at the endosome and cycles between the cytosol and the endosomal membrane, just like its interaction partner CHMP4B. They also indicate that CC2D1A appears to be removed from the endosomal membrane by VPS4 and suggest that CC2D1A is involved in ESCRT-III function in mammals.

Unfortunately, we were unable to confirm an endogenous endosomal localisation for CC2D1B, as our antibody was not specific in histological staining. Nevertheless, over-expressed CC2D1B also accumulated on the enlarged vesicles in *Vps4a* ^*+/-*^,*Vps4b* ^*+/-*^ cells (S5C Fig), suggesting that CC2D1A and CC2D1B are temporally associated with the endosomal membranes and are involved in ESCRT function.

### Endogenous interaction between CC2D1A and CHMP4B

**Fig 8:**
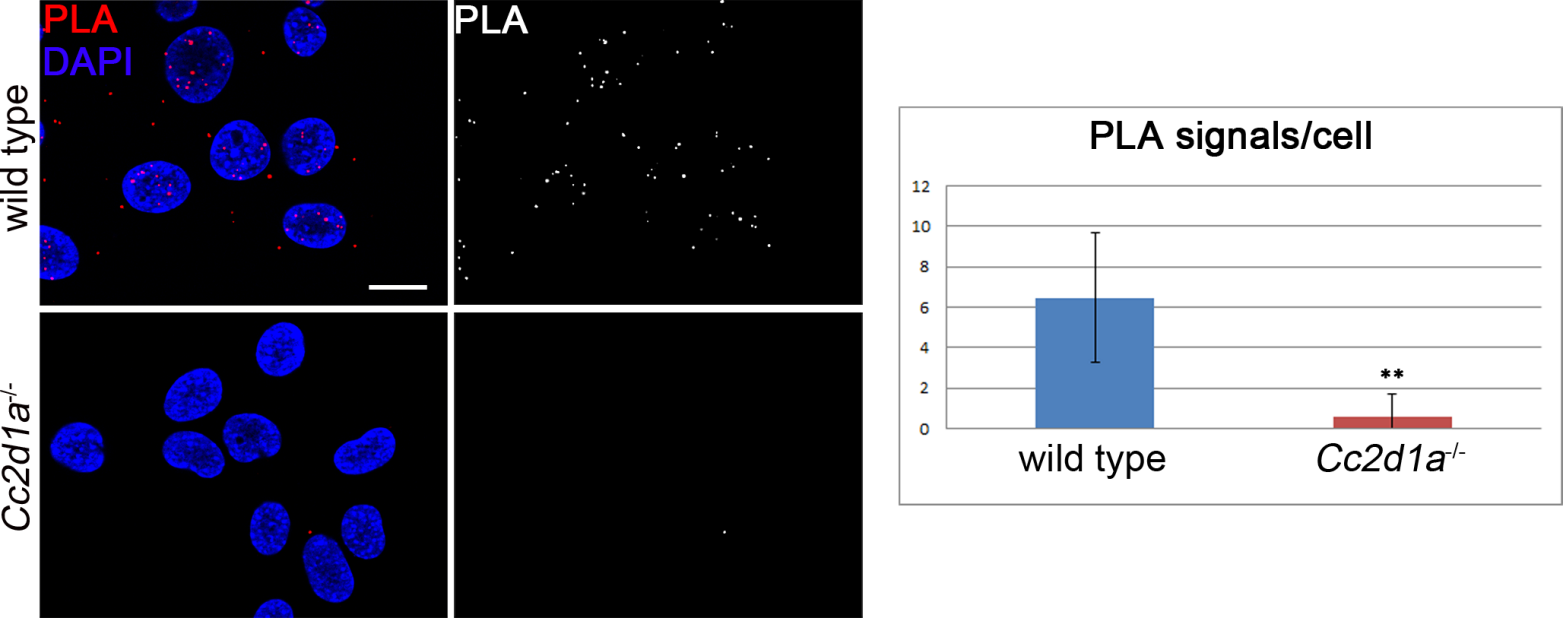
Endogenous CC2D1A and CHMP4B interact in wild type MEFs. PLA (Proximity Ligation Assay) was performed on wild type and *Cc2d1a* deficient MEFs. Positive signals were abundant only in wild type cells. Data are mean ± SD values from 2 independent experiments (** p < 0.001). Scale bars are 20 μm.

So far only *in vitro* data showed physical interactions of CHMP4B with CC2D1A. In order to test whether this can also be observed between the corresponding endogenous proteins in a cell, we performed the proximity ligation assay (PLA). PLA enables the *in situ* detection of endogenous protein protein interactions, using protein specific antibodies [47]. For the assay, we compared wild type and *Cc2d1a* deficient MEF cells and used our specific CC2D1A antibody in combination with a commercially available CHMP4B antibody. As expected, only few PLA signals were visible in *Cc2d1a* deficient cells (0.6±1.07), confirming the accuracy of the method (Fig 8). In contrast, abundant PLA signals were detected in wild type MEFs (6.47±3.18) indicating that endogenous CC2D1A and CHMP4B interact (Fig 8). To exclude the possibility that CHMP4B and CC2D1A interact in the cytosol randomly by freely diffusing cytosolic proteins we performed PLA with CHMP4B and the cytosolic protein PGK1 (S6 Fig). Again, we could detect a clear interaction only in the control and not between CHMP4B and PGK1 (5.61±2.39 for CHMP4B with CC2D1A compared to 0.64± 1.01 for CHMP4B with PGK1). Taken together, these experiments further support the assumption that CC2D1A and possibly also CC2D1B are involved in ESCRT-III mediated events.

### Deficiency of *Cc2d1a* leads to enlarged endo/lysosomes

**Fig 9:**
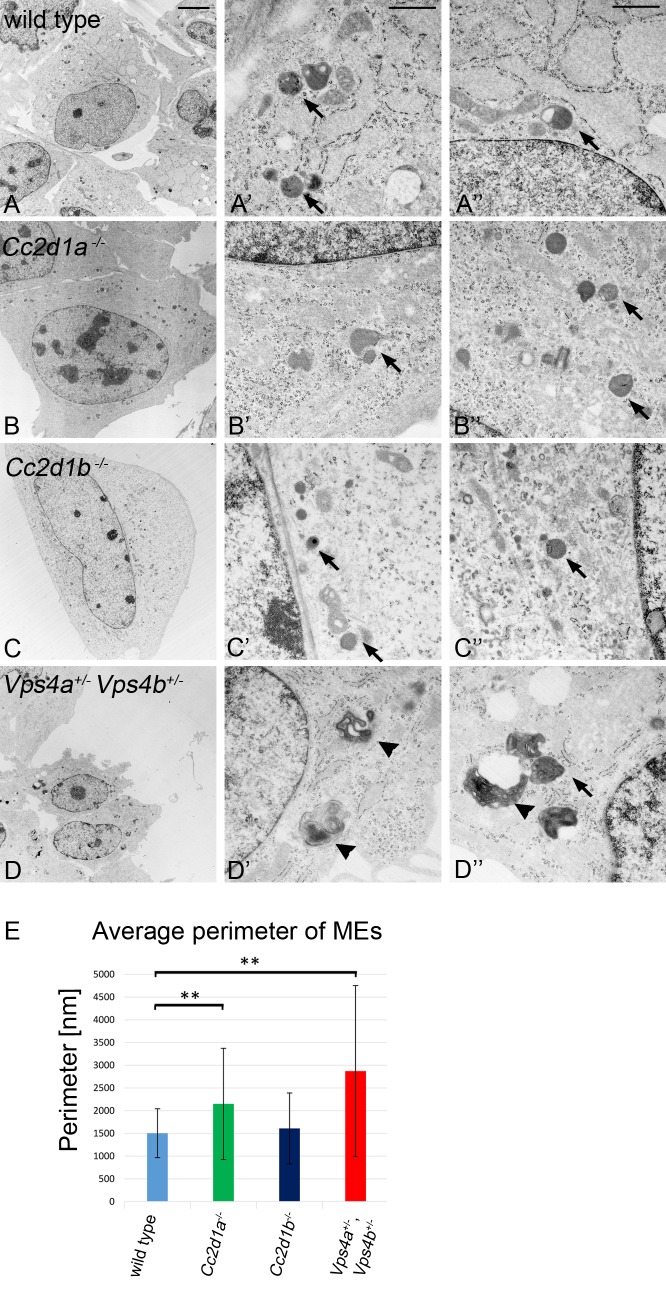
Ultra-structural characterisation of endo/lysosomal compartments in wild type, *Cc2d1a* ^-/-^, *Cc2d1b* ^-/-^ and *Vps4a* ^+/-^,*Vps4b* ^+/-^ MEFs. (A-A”) wild type, (B-B”) *Cc2d1a* ^-/-^ mutant, (C-C”) *Cc2d1b* ^-/-^ mutant and (D-D”) *Vps4a* ^+/-^,*Vps4b* ^+/-^ double heterozygous MEFs. The comparison reveals that *Cc2d1a* ^-/-^ and *Cc2d1b* ^-/-^ contain endo/lysosomal organelles that are similar to wild type endo/lysosomal organelles in appearance. In contrast, the appearance of the organelles is dramatically changed in *Vps4a* ^+/-^,*Vps4b* ^+/-^ double heterozygous cells. These cells contain massively enlarged MEs with a class E like phenotype. The MEs partially lose their normal round shape and contain many intraluminal vesicles or membrane layers (arrowheads). (E) Statistical analysis of the endo/lysosomal perimeter revealed that the organelles in *Cc2d1a* ^-/-^ and *Vps4a* ^+/-^,*Vps4b* ^+/-^ MEFs are significantly enlarged compared to wild type cells. Data are mean ± SD values from 4 independent experiments (** p < 0.001). Arrows highlight individual endosomes. Scale bars are 5 μm (*A-D*) and 0.5 μm (*A*`-*D*`, *A*”-*D*”).

To further elucidate a function of CC2D1 proteins in endosome maturation we analysed the endo/lysosomal compartments of wild type and mutant MEFs with the transmission electron microscope (TEM) (Fig 9). Endo/lysosomal organelles were identified by their ultrastructural characteristics proposed by others [48–50]. Exemplary pictures of the analysed endo/lysosomal organelles are depicted in S7A–S7D Fig. *Cc2d1b* ^-/-^ cells show no differences in size or morphology of the endo/lysosomal compartments compared to wild type cells (Figs 9A–9A”, 9C–9C”, 9E and S7E). In contrast and in accordance with the fluorescence microscopy data, *Vps4a* ^+/-^,*Vps4b* ^+/-^ cells had enlarged endo/lysosomal compartments. We detected large multi-membrane structures that resembled the class E compartment described for loss of ESCRT function in yeast and mammalian cells [51,52]. We did not observe these structures in *Cc2d1a* ^-/-^ cells, but found that the average size of their endo/lysosomal organelles was significantly increased (Figs 9B–9B”, 9E and S7E). The endo/lysosomal phenotype of *Cc2d1a* ^-/-^ cells resembled that observed in *lgd* mutant cells of *D*. *melanogaster* [53]. When we allocated the endo/lysosomal organelles to different size classes, we found classes in *Cc2d1a* ^*-/-*^ and *Vps4a* ^+/-^,*Vps4b* ^+/-^ MEFs that were not present in *Cc2d1b* ^-/-^ or wild type MEFs (S7E Fig). Thus, the loss of *Cc2d1a* function results in a moderate enlargement of the endo/lysosomal compartment, indicating that it is involved in endosomal maturation. Moreover, already the double heterozygosity of *Vps4a* and *Vps4b* results in a dramatic morphological defect of the endo/lysosomal phenotype, which appears to have no direct consequences for the viability of mice in captivity.

### Human CC2D1A and CC2D1B can replace the function of Lgd in *Drosophila melanogaster*

In order to obtain further evidence for the conservation of the function of the Lgd proteins in metazoans, we asked whether and to what extent the two mammalian orthologs of Lgd could replace the loss of function of *lgd* in *D*. *melanogaster*. Since it turned out that rescue experiments with the Gal4/UAS system are not suitable [22], our previously reported results are not meaningful [1]. We therefore here used an assay that we have later developed for the structure-function analysis of Lgd [22]. In short, human CC2D1A and CC2D1B were cloned behind the *lgd* promoter (lgdP) and the constructs were inserted into the same landing site to neutralise position effects on expression. Thus, CC2D1A and CC2D1B are expressed at endogenous level and similar to each other. This allowed the direct comparison of the effects caused by the two constructs.

**Fig 10:**
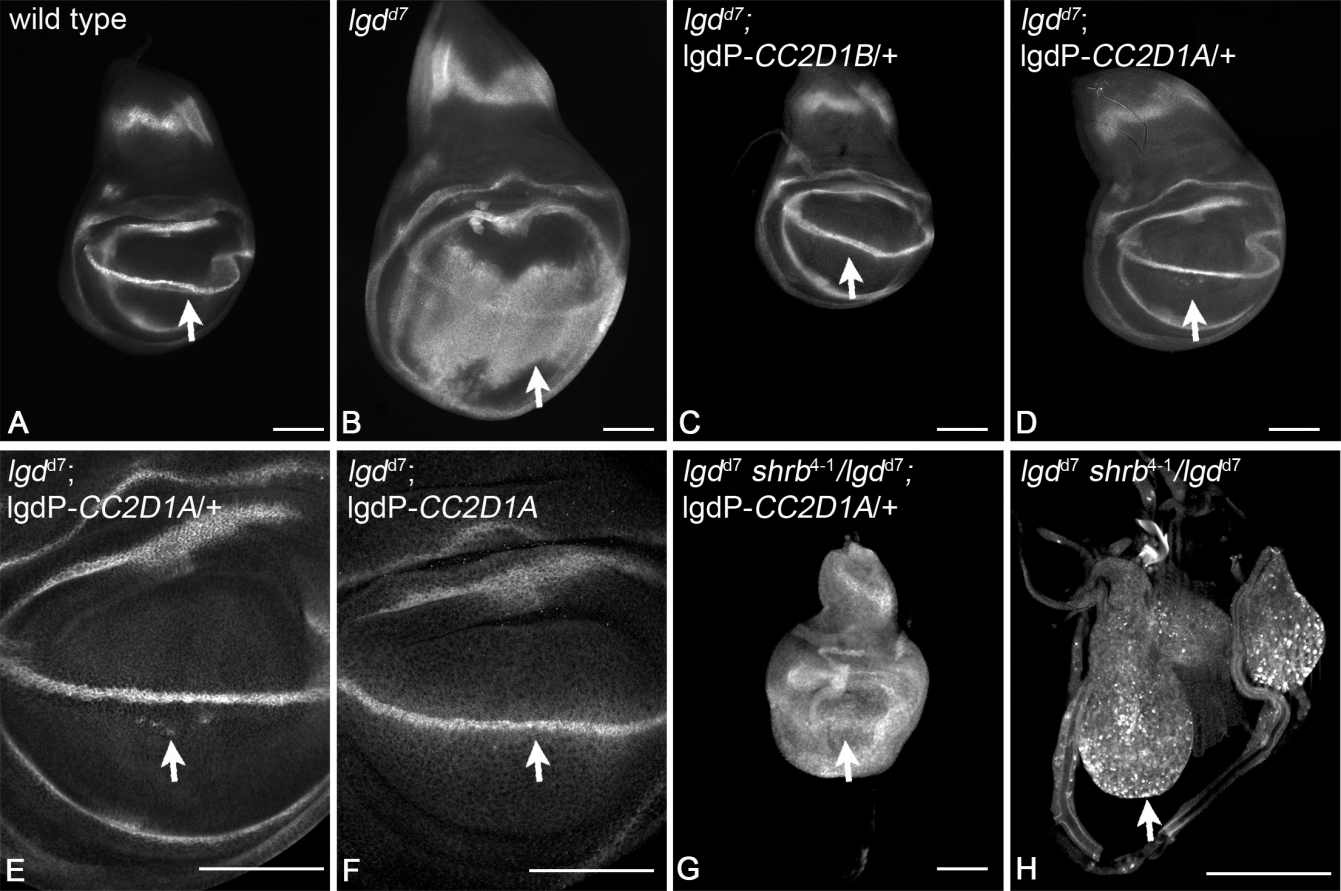
Human CC2D1A and CC2D1B can replace the function of Lgd in *D*. *melanogaster*. (A) Expression of the Notch target gene *wingless* (Wg) in a wild type wing imaginal disc. (B) Expression of Wg in an *lgd* null mutant disc (lgd^d7^). The expression domain has broadened dramatically, indicating an expansion of Notch activity. In addition, the disc is much larger due to over-proliferation of the disc cells. Expression of one copy of CC2D1B (C) or CC2D1A (D) normalises the expression of Wg. (E) Magnification of the wing area of the disc shown in (D). It reveals that a few cells still express Wg ectopically indication that the rescue of *lgd* mutants by CC2D1A is incomplete. (F) The ectopic expression of Wg is completely suppressed if two copies of CC2D1A are present in the genome. (G) The phenotype of *lgd* mutant discs rescued with one copy of CC2D1A worsened dramatically upon loss of one copy of *shrb*. (H) The wing imaginal disc of an *lgd* mutant, *shrub* heterozygous animal. The animals die during the early third larval instar stage and contain very small imaginal discs with Wg primarily located in dramatically enlarged endosomes. Arrows point to the expression domain of Wg along the dorsoventral boundary, which is controlled by the activity of the Notch pathway. Scale bars are 100μm.

Loss of *lgd* function in *D*. *melanogaster* results in over-proliferation of the imaginal discs, which are epithelial monolayers (Fig 10A and 10B). Moreover, target genes of Notch such as *wingless* (*wg*) are ectopically expressed, indicating that the Notch pathway is ectopically activated ([2], Fig 10A and 10B). We found that both orthologs could rescue the *lgd* mutant phenotype, albeit to different extent (Fig 10C–10F). *lgdP*-*CC2D1B* completely rescued the mutant, even if only one copy was present in the genome (Fig 10C). We obtained normal looking fertile flies. In the case of *lgdP*-*CC2D1A* only two copies present resulted in a complete rescue of the imaginal disc phenotype (Fig 10D–10F). However, in this case the fully differentiated flies failed to hatch. These experiments reveal functional differences between the human CC2D1 proteins for the first time and indicate that CC2D1B is more similar to Lgd than CC2D1A. Nevertheless, both orthologs can rescue the *lgd* mutant phenotype to different extent, indicating a significant overlap in their function.

The rescue of *lgd* mutants with only one copy of *lgdP*-*CC2D1A* resembled that of weak *lgd* mutants [1]. We have previously shown that the phenotype of *lgd* mutants dramatically worsened if one copy of *shrb* is additionally removed (*shrb* heterozygosity) [22], indicating a functional relationship between the two loci (Fig 10H). We used this test of genetic interaction between *shrb* and *lgd* to test whether also CC2D1A requires the interaction with Shrb in *D*. *melanogaster*. We found that the phenotype of *lgd* ^*d7*^;*lgdP*-CC2D1A also dramatically worsened if one copy of *shrb* was removed (Fig 10G; genotype: *lgd* ^d7^ *shrb* ^4-1^/*lgd* ^d7^; *lgdP*-*CC2D1A*/+). This genetic interaction suggests that Shrb functionally interacts with CC2D1A in a similar manner as we have found for Lgd [22]. Our results suggest for the first time that the CC2D1 proteins functionally interact with members of the CHMP4 family in an organism.

## Discussion

Here we report the first characterisation of the Lgd homologue CC2D1B in a mammalian system. We found that it is not an essential gene in mouse, since its loss of function results in no detectable phenotype. We confirmed the previously reported finding that the loss of function of its paralogue CC2D1A results in postnatal death due to failure to breathe [15,17,21]. We here extend this finding by showing that the postnatal death is mostly caused by loss of its function in the nervous system. This result extends previous studies, that suggested either an involvement of CC2D1A in neuronal differentiation and brain development via the regulation of the Protein Kinase A [15] or in functional maturation of synapses [21]. Thus, a defect in certain neurons or glia cells is probably the cause of the breathing defect and the resulting death of mutant animals.

We failed to find evidence for an involvement of the CC2D1 proteins in cell division. Instead *Cc2d1a* or *Cc2d1b* mutants develop normally and MEFs harvested from these homozygous mutants do not show a cell division phenotype nor are they multi-nucleated as has been found for HeLa cells treated with *CC2D1A* specific siRNA [18]. This is in agreement with the fact that mutant animals develop until birth with no obvious defects.

In *D*. *melanogaster* the loss of function of *lgd* results in the constitutive ligand-independent activation of Notch in several epithelia [1–4,6]. In order to test whether the loss of one of the orthologs results in ectopic Notch activation in mammals, we analysed their function in the epithelium of the gut of mice. In contrast to *D*. *melanogaster*, we did not find any evidence for ectopic activation of the Notch signalling pathway if one of the two orthologs is inactivated. The number or distribution of goblet cells did not change and the expression of the Notch activity reporter Hes1-emGFP and endogenous *Hes1* was unaffected upon loss of function of *Cc2d1a* or *Cc2d1b*. This suggests that the individual loss of the genes does not result in uncontrolled activation of the Notch pathway in mammals. Notch signalling is critical for T cell development (reviewed by [54]). In *Cc2d1a* deficient embryos the development and function of thymocytes is not impaired [21] further supporting the notion that Notch signalling is not altered in the absence of *Cc2d1a* alone.

It might still be possible that weak ectopic activation of the Notch pathway occurs in mutant mice, but this activation is too weak to be detected with our test systems and to manifest itself in phenotypes in the gut epithelium. However, it is possible that this weak activity has effects in other tissues, such as the brain. Indeed, it has been reported that CC2D1A over-expression leads to weak Notch reporter activity in HEK293 cells [20]. This weak activation might be responsible for the brain defect, since the Notch pathway is known to regulate neural development and postnatal neurogenesis (reviewed by [55]). Moreover, activation of the pathway in cultured neurons changes their dendritic arborisation [56,57]. Importantly, a similar arborisation defect together with activation of the Notch pathway has been observed upon inactivation of Shrub, the ortholog of the CHMP4s in *D*. *melanogaster* [27]. However, CC2D1A was also shown to act as a positive regulator of the cAMP/PKA pathway [14,15] that is known to regulate synaptic plasticity, learning and memory [58,59] and the NF-κB pathway [7] that is involved in the regulation of neuronal differentiation and survival [60]. Intriguingly, NF-κB activation was shown to be specific to CC2D1A, while CC2D1B could not activate NF-κB [20] suggesting that the proteins are not functional redundant with regard to NF-κB signalling. These finding could also explain why *Cc2d1a* deficient mice die early while *Cc2d1b* deficient mice display no obvious phenotype.

Our pulse and chase experiment to analyse the endosomal degradation of the human NOTCH1 receptor showed no alterations in wild type and *Cc2d1a* deficient MEFs. Nevertheless, we cannot rule out the possibility that our method might not be sensitive enough for detection if a slight delay or defect in degradation occurs. The siRNA mediated depletion of *CC2D1A* in HeLa cells, analysed with high-resolution confocal microscopy and quantitative multiparametric image analysis, detected a decrease in EGF and TF endocytosis [29], supporting a function of CC2D1A in the endocytic pathway. Indeed we found clear evidence for an involvement of CC2D1 proteins in the endocytic pathway: 1. Our EM analysis revealed that the endosomes are enlarged upon loss of *Cc2d1a* function. Importantly, the classification according to size revealed that larger size classes appear in the mutant that are absent in the wild type. This has also been found *lgd* mutant cells in *D*. *melanogaster* [53]. 2. We found that a fraction of both CC2D1 proteins is associated with membranes. 3. While CC2D1A is distributed in the cytosol in wild type MEFs, it accumulates on endosomal membranes of *Vps4a* ^+/-^,*Vps4b* ^+/-^ double heterozygous MEFs. This relocation is striking and suggests that, like the CHMP4 proteins, CC2D1A and probably also CC2D1B cycles between the cytosol and the limiting membrane of the endosome. It also suggests that both proteins are involved in the function of ESCRT-III. 4. We show that CC2D1A interacts with CHMP4B at endogenous level. This has not been shown for the full-length proteins and especially not under endogenous conditions. Thus, our findings show for the first time that this interaction occurs *in vivo*. 5. We show that both CC2D1 proteins can replace the function of Lgd in *D*. *melanogaster*. In the fly Lgd is evidently involved in endosomal trafficking of transmembrane proteins and both Lgd orthologs can take over this function. Moreover, CC2D1A genetically interacts with the CHMP4 ortholog Shrub in *D*. melanogaster, suggesting that it requires Shrub respectively the CHMP4 orthologs for its function. Interestingly, the rescue ability of both orthologs differed: While the imaginal disc phenotype could be completely rescued by both CC2D1 proteins, only flies expressing CC2D1B were viable and fertile. CC2D1A contains an extended C-Terminus with no putative domains that is missing in Lgd and CC2D1B. This C-terminal extension might be the reason for the poorer rescue ability and might be required for the suggested additional non-redundant functions of CC2D1A in mammals.

Despite the differences in the extent of the rescue, our experiments in *D*. *melanogaster* suggest that CC2D1A and CC2D1B have overlapping functions in the endocytic pathway. Thus, it is likely that functional redundancy among both CC2D1 proteins prevents the formation of a detectable cellular phenotype if only one gene is deleted, especially since we found that both proteins are present in all cells tested. Therefore, it will be necessary to delete both genes to further evaluate their function in the endocytic pathway and Notch pathway regulation. The analysis of these double mutants will illuminate the function of CC2D1A and CC2D1B in mammals.

## Materials and Methods

### Generation of a *Cc2d1a* conditional knockout mouse

The *Cc2d1a* targeting vector was generated using the pGK12 vector (Artemis Pharmaceuticals, Köln) which contains a PGK *neomycin* cassette, as a positive selection marker, and the *Herpes Simplex virus thymidine kinase* (TK) gene, as a negative selection marker. The 2.3 kb 5`arm, the deleted area (4kb, exons 7–14) and the 5.0 kb 3`arm were amplified via PCR using BAC-DNA RP23-298K21 (BACPAC Resources, Oakland, USA) as a template and confirmed by sequencing. The 129/B6 F1 hybrid ES cell line v6.5 [61] was electroporated with the *Not*I linearized targeting vector. Using Southern Blot analysis five targeted ES cell clones were identified and injected into blastocysts of CB20 mice and transplanted into pseudopregnant host mothers. The resulting chimeric mice were bred to C57BL/6N mice. Resulting *Cc2d1a* ^neoflox/+^ animals were crossed with FLPe-Deleter mice [62] to delete the FRT-flanked *neomycin* resistance cassette (*Cc2d1a* ^flox/+^ mice). Heterozygous *Cc2d1a* ^flox/+^ siblings were crossed to generate homozygous *Cc2d1a* ^flox/flox^ mice that are viable, fertile and do not display any obvious phenotype. For a full knockout Cc2d1a^neoflox/+^ animals were crossed with Cre-Deleter mice [63]. Resulting *Cc2d1a* ^+/-^ siblings were crossed to generate homozygous *Cc2d1a* ^-/-^ mice.

### Generation of a *Cc2d1b* conditional knockout mouse

The mouse strain C57BL/6N-*Cc2d1b* ^tm1a(KOMP)Wtsi^ used for this research project was created from ES cell clone EPD0017_2_C11 obtained from the NCRR-NIH supported KOMP Repository (https://www.komp.org/) and generated by the CSD consortium for the NIH funded Knockout Mouse Project (KOMP). Methods used on the CSD targeted alleles have been published elsewhere [64]. *Cc2d1b* ^tm1a(KOMP)Wtsi^ mice were crossed with C57BL/6-TgN(FLPe) mice [62] to obtain the conditional allele *Cc2d1b* ^flox^. *Cc2d1b* ^flox/flox^ animals do not display any obvious phenotype and are fertile. For a complete knockout, *Cc2d1b* ^flox/flox^ animals were crossed with C57BL/6;129/Ola-TgH(Deleter+8)KP animals [63] to delete the floxed exon to generate a frameshift mutation. Resulting *Cc2d1b* ^+/-^ siblings were crossed to generate homozygous *Cc2d1b* ^-/-^ mice.

### Generation of *Vps4a* and *Vps4b* knockout mice

The C57BL/*Vps4a* ^+/-^ heterozygous knockout mouse strain resulted from a conditional *Vps4a* knockout model. Vector construction and targeted knockout strategy was designed together with genOway (Lyon, France), where mice were generated. 129Sv/Pas ES cells were transfected with the targeting construct pVPS4a-KOE1-HR. This vector contains a short (1,9 kb) and a long (5,8 kb) *Vps4a* homology region. Two *Lox*P sites were inserted flanking *Vps4a* exon 2 and exon 3. The positive selection neomycin gene flanked by FRT sites is inserted upstream of exon 2 and the negative selection marker Diphteria Toxin A (DTA) is located downstream from the distal long homology arm at the 3’ end of *Vps4a*. Homologous recombination in ES cells was demonstrated by PCR and Southern blot analysis. After blastocyst injection male chimeras were mated with female Flp-expressing mice (C57BL/6J) to excise the neomycin selection cassette and to generate heterozygous mice carrying the floxed *Vps4a* allele. Breeding floxed *Vps4a* mice with C57BL/6J Cre-expressing mice led to constitutive heterozygous *Vps4a* knockout mice lacking exon 2 and 3. The heterozygous *Vps4a* knockout mice were inbred with C57BL/6 mice for more than 10 generations.

The 129Sv/*Vps4b* ^+/-^ is a classical knockout mouse strain that was generated using the targeting construct pVPS4b-KO, where the *Vps4b* exon 1–4 region is replaced by a neomycin-resistance cassette. The negative selection marker DTA is located at the 3’ end of the *Vps4b* homology region. Following homologous recombination in ES cells and blastocyst injection, heterozygous *Vps4b* knockout mice were confirmed by Southern blot analysis and inbred with 129Sv mice for more than 10 generations. This *Vps4b* knockout mouse strain was generated in collaboration with U. Rüther (Düsseldorf).

A more detailed description of the experimental steps will be published elsewhere together with a comprehensive phenotype analysis of conditional and constitutive *Vps4a* and *Vps4b* mice.

### Mouse strains

Nestin-Cre mice (C57BL/6J.B6SJF2-TgN(Nestin-Cre)) have been described elsewhere [39] and were a kind gift from R. Kühn. Villin-Cre mice (B6.SJL-Tg(Vil-cre)997Gum/J) were purchased from The Jackson Laboratory (stock number 004586). Hes1-emGFP^SAT^ mice were a kind gift from S. Fre [42]. C57BL/6-TgN(FLPe) mice have been described elsewhere [62] and were a kind gift of A. Gödecke. C57BL/6;129/Ola-TgH(Deleter+8)KP mice have been described elsewhere [63] and were a kind gift of K. Pfeffer.

Mice were maintained in the central animal research facility of the Heinrich-Heine-University Duesseldorf under specific pathogen-free conditions. Animals got food and water *ad libitum*.

All experiments performed on animals in this study were approved by the Animal Care and Use Committee of the local Government of Düsseldorf in accordance with the German law for animal protection and were carried out with the authorization of the LANUV (Landesamt für Natur-, Umwelt- und Verbraucherschutz) of North-Rhine-Westphalia, Germany under the reference number 9.93.2.10.31.07.249.

### PCR genotyping of mice

Genomic DNA was isolated from tail biopsis using DirectPCR Tail buffer (Viagen) and PCR was carried out using 1 unit of Crimson Taq DNA polymerase (NEB) in a 25 μl standard reaction mix. Oligonucleotide sequences for genotyping are as follows: *Cc2d1a*: P1: 5`agaccctgtggctggattgt3`, P2: 5`acccatcctttgcttgtctc3`, P3: 5`gccagcctggtctacaatca3`and P5: 5`cctgacctgagtactggaca3`. *Cc2d1b*: 1F: 5`gcatgtgccacaatgccaagc3`, 3R: 5`ctgagtgagcagttcctagc3`and 3bR: 5`aggctgcctctaagggttcc3`. Nestin-Cre: p-CRE1: 5`atgcccaagaagaagaggaaggt3`, pCRE2: 5`gaaatcagtgcgttcgaacgctaga3`, oIMR7338: 5`ctaggccacagaattgaaagatct3`and oiMR7339: 5`gtaggtggaaattctagcatcatcc3`. Villin-Cre: P1878: 5`gtgtgggacagagaacaaacc3`, P1879: 5`acatcttcaggttctgcggg3`, P8744: 5`caaatgttgcttgtctggtg3`and P8745: 5`gtcagtcgagtgcacagttt3`. Hes1-emGFP^SAT^: P35: 5`cccaagttcgggtgaaggc3`and P36: 5`ccttggacaatgccacccaa3`. Vps4a: Vps4a-5F: 5`tataatatggttgagcctcccttc3`, 3.1 Rev Vps4a: 5`gcaccccaaactggaaaaccacttactctcc3`, Vps4a-5R: 5`attcgtgacctatctcgattcttc3`. Vps4b: Vps4bKO_NEO_For1: 5`aggattgggaagacaatagcag3`, Vps4b_WT_For2: 5`tgctttgaggaactaaatcatcc3`, Vps4b_WT_Rev2: 5`ggattggactcaatgcctacat3`.

### Generation and purification of antibodies

For the production of a CC2D1B/mLGD1 antibody a murine *Cc2d1b* cDNA fragment (covering amino acids 1–253) was amplified from RIKEN Mouse FANTOM Klon A830039804 with the primers mLGD1-AK-For (5`aaactcgaggcatgccagggccaagacc3`) and mLGD1-AK-Rev (5`aaagcggccgctggctccatggcacaggga3`) and cloned into the GST affinity tag containing vector pGEX-6P-2 (GE Healthcare Bio-Sciences) using *Xho*I and *Not*I restriction sites. The GST-CC2D1B(1–253) fusion protein was expressed in *E*. *coli* and captured by immobilized glutathione. After removal of GST affinity tag by PreScission Protease guinea pigs were immunized with the CC2D1B(1–253) antigen (Cocalico Biologicals Inc.). Serum from the final bleed was further affinity purified with the antigen immobilized on nitrocellulose membrane.

For the production of a CC2D1A/mLGD2 antibody guinea pigs were immunized (Eurogentec s.a.) with a GST tagged antigen that consists of the 4^th^ DM14 domain (amino acids 481 to 640) fused to the extended C-Terminus of CC2D1A (amino acids 788 to 943) that is absent in CC2D1B. For this two cDNA fragments (477–647 and 788–943) were amplified using the primer pairs mLGD2-481-For(5`aggatccaacagagcccagcagcagct3`)/mLGD2-640-Rev(5`acccgggcttgatgacactgaaggtcctctg3`) and mLGD2-788-For(5`acccggggcccagcagttggaaactacaac3`)/mLGD2-943-Rev(5`actcgagtcacctgcggagtcgctgca3`) and cloned into pGEM-T Easy vector (Promega). Ligation of *Sma*I and *Dra*III digested fragments led to fusion of both cDNA fragments. Using *BamH*I and *Xho*I digestion the cDNA was cloned into the GST affinity tag containing vector pGEX-6P-2 (GE Healthcare). The GST-CC2D1A(477–647,788–943) fusion protein was expressed and purified by immobilized glutathione before the immunization of guinea pigs (Eurogentec s.a.). Serum from the final bleed was further affinity purified with the antigen immobilized on nitrocellulose membrane.

### Immunoblotting analysis

After homogenisation and short sonication of tissue in lysis buffer (20 mM Tris-HCl pH 7.4, 100 mM NaCl, 10% Glycerol, 0.5% Triton X-100, proteinase inhibitor cocktail (Roche, 1:200) and 1 mM PMSF), the lysates were centrifuged at 20,000 x g for at least 20 minutes at 4°C. The soluble fraction was collected and loaded onto 8–10% SDS-PAGE gel and immunoblotted according to standard protocols. The following antibodies were used: Actin (Sigma, A-5060, 1:5000), alpha-Tubulin (Sigma-Aldrich, Clone B-5-1-2, 1:10000), CC2D1A (Abnova, H00054862-BO1P, 1:2000), CC2D1B (Proteintech, 1:4000), CHMP4B (Santa Cruz C12, 1:2000), LAMP1 (DSHB, 1D4B, 1:2000) and PGK1 (Gene Tex, GTX107614, 1:500). For the quantification of protein levels CC2D1A/mLGD2 (this study, 1:500) and CC2D1B/mLGD1 (this study, 1:1000) antibodies were used. Relative protein levels were determined using ImageJ.

### RT-PCR and qRT-PCR

Total RNA was isolated from tissues using TriFast Reagent (Peqlab). 1–3 μg of isolated RNA was treated with DNase I and then reverse-transcribed using the SuperScript First-Strand Synthesis System (Invitrogen by Life Technologies) or the GoScript Reverse Transcription System (Promega) according to manufacturers`instructions.

RT-PCR was carried out with 0.5 μl of cDNA, 0.2 μM each of forward and reverse primers, 200 μM dNTPs and 0.5 units of Crimson Taq DNA polymerase (NEB) in a 25 μl reaction. Cycling conditions were as follows: 35 cycles of 94°C (30 s), 56°C (30 s), and 72°C (30 s). mHPRT (hypoxanthin-phosphoribosyl-transferase) was used as internal control. Oligonucleotide sequences for RT-PCR are as follows: mHPRT-for: 5`cgtcgtgattagcgatgatg3`, mHPRT-rev: 5`tatgtcccccgttgactgat3`. CC2D1A-Ex2-F: 5`gggattaatgaggaggagctg3`, CC2D1A-Ex4-R: 5`gccttctgttcttctccaagg3`, CC2D1A-Ex12-F: 5`tagtgggtgtcctggaaactg3`, CC2D1A-Ex13-R: 5`gtcctgatggaggtgctttg3`, CC2D1A-Ex22-F: 5`agatcctggaggttttggatg3`, CC2D1A-Ex25-R: 5`caacacgctaaggctatgcag3`, CC2D1B-Ex1-F: 5'gagaaggcccggttttggtt3', CC2D1B-Ex4-R: 5'tacagtctgctgccagcttc3', CC2D1B-Ex16-F: 5'aggaggtgtatgcccagcta3', CC2D1B-Ex17-R: 5'cgagtggtctcagccacatt3', CC2D1B-Ex24-F: 5'ctgagaactggctggtcctg3' and CC2D1B-Ex25-R: 5'acagagcagtggggtatcct3'.

Quantitative analysis of *Hes1* gene expression in the murine intestinal epithelia was performed by quantitative RT-PCR using a Stratagene Mx3005P cycler (Agilent Technologies), with KAPA SYBR FAST qPCR Kit Master Mix (2x) Universal (Peqlab) using 1 μl cDNA according to manufacturer´s instructions. *Gapdh* (glyceraldehyde-3-phosphate dehydrogenase) expression was used as an internal control. Oligonucleotide sequences for quantitative RT-PCR were as follows: mHes1-F: 5`accccagccagtgtcaaca3`, mHes-R: 5`tgtgctcagaggccgtctt3`, mGapdh-F: 5`tgaaggtcggtgtgaacgg3`and mGapdh-R: 5`cgtgagtggagtcatactggaa3`. The relative expression levels of *Hes1* mRNA in the intestinal epithelia were calculated and analyzed by the 2-ΔΔCT method. Statistical analysis was performed using Student`s t-test, unpaired, two-tailed.

### Histology and immunohistochemistry

For cryopreservation tissues were fixed in Stefanini`s Fixative (2% PFA, 0.2% Picric acid) over night, treated with 30% Sucrose solution (up to 12 hours) and were embedded in Tissue Tek (Sakura Finetek). Embedded tissues were cut on a cryostat into 5–7 μm thick slices and transferred to Superfrost slides. For antigen retrival sections were boiled in 10 mM citrate buffer (pH 6.0) for 10 min. Sections were then washed 3 times in dPBS, permeabilised for 10 min in 0.3% Triton X-100 in dPBS and then incubated with a 10% blocking solution (10% NGS, 0.1% Tween-20, 0.3 M Glycine in dPBS) for 1 hour at room temperature. Sections were incubated with primary antibodies (Ki67 (abcam, ab16667, 1:200) and GFP (Roche, 11814460001, 1:500) in a 1.5% blocking solution (1.5% NGS, 0.1% Tween-20 in dPBS) overnight at 4°C. Sections were then rinsed three times in dPBS and subsequently incubated with secondary antibodies (1: 500 in 1.5% blocking solution) for 1 hour at room temperature. After that, sections were washed in dPBS, the nuclei were counterstained with DAPI (0.3 μg/ml) and mounted with VECTASHIELD (Vector Laboratories). Alcian blue staining was performed according to standard protocols on paraffin sections. For the quantification of goblet cell number, the number of stained cells in 250μm of a crypt-villus unit (35–46 units/animal, n = 3) were counted. Statistical analysis was performed using Student`s t-test, unpaired, two-tailed.

### Constructs

Human CC2D1A cDNA was amplified from clone 6585236 (Source BioScience) with the oligonucleotides dsRed-CC2D1A-For (5`aactcgagtggaattcgccatgcacaag3`) and dsRed-CC2D1A-Rev (5`aaaagcttagcgtaatctggcacatcg3`) and cloned into pDsRed-Monomer-N1 vector (Clontech) using *Hind*III and *Xho*I restriction sites. Human CC2D1B cDNA was synthesised (from Ensembl transcript ENST00000284376, GenScript) and was amplified with the oligonucleotides EGFP-CC2D1B-For (5`aagaattcgcggcggcccatgatgc3`) and EGFP-CC2D1B-Rev (5`atctagcatgctcgagtc3`) and cloned into pEGFP-C1 (Clontech) using *Xho*I and *EcoR*I restriction sites. *VPS4B* cDNA was amplified from HeLa cDNA using the oligonucleotides VPS4-For (5`agaattcatgtcatccacttcgcccaacc3`) and VPS4-Rev (5`agcggccgcgccttcttgaccaaaatcttc3`) and cloned into pcDNA3 vector with an N-terminal Myc Tag (Invitrogen) using *EcoR*I and *Not*I restriction sites. The dominant negative VPS4B was generated by a site-directed mutagenesis PCR using the oligonucleotides VPS4B-E235Q-For (5`ccctccattatcttcattgatcaaattgattctc3`) and VPS4B-E235Q-Rev (5`ccacagagagaatcaatttgatcaatgaagataatggaggg3`) and pcDNA3-VPS4B-Myc as a template according to standard protocols.

### Cell culture

MEFs were isolated from E13.5-E15.5 mouse embryos according to standard protocols. After 2–3 passages cells were transfected with pMSSVLT SV40 vector containing the SV40 Large T Antigen and immortalized cells were selected by G418 treatment.

HeLa and MEFs were maintained in DMEM supplemented with 10% FCS, Pen/Strep (50 Units/ml) and, for immortalized cells with G418 (0.5 μg/ml), and cultured at 37°C in a humidified incubator with 5% CO_2_. All transfections were done with Lipofectamine 2000 (Life Technologies) according to the manufacturer`s instructions.

The cell proliferation assay was performed with CellTiter 96 AQueous Non-Radioactive Cell Proliferation Assay (Promega) according to the manufacturer`s instructions. For the calculation of the mitotic index 1000–1500 cells of two to three different passages per genotype were counted and the number of cells undergoing mitosis (visualized by DAPI staining) was divided by total cell number and multiplied by 100.

### Cell fractionation

Cells (three 10 cm dishes) were cultured to confluence, washed in dPBS, harvested by scraping and centrifuged at 600 x g for 5 min and homogenised in 1 ml buffer containing 20 mM HEPES-KOH, pH 7.2, 400 mM sucrose and 1 mM EDTA by passing through a 22 G needle. Homogenates were centrifuged at 1,000 x g for 3 min at 4°C to pellet cell debris and nuclei. Supernatants were centrifuged at 100,000 x g (Ti 70.1 rotor, Beckmann) for 45 min at 4°C. The supernatant contained the cytoplasm fraction and the pellet the membrane fraction, which was resuspended in 500 μl homogenisation buffer. The protein concentration of each fraction was determined via Bradford assay and equal amounts of protein were used for Western blot analysis.

### HA antibody uptake assay

HA-tagged NOTCH1 expressing MEFs were generated by retroviral transduction according to standard protocols. Briefly, Plat-E ecotropic packaging cell line was transfected with pBABE vector containing human NOTCH1-HA (kind gift of J. C. Aster [43]) and virus supernatant was then used to infect MEFs. Infected cells were identified by puromycin selection and clonal populations were obtained by dilution. For the uptake assay cells were briefly washed in serum-free DMEM and then incubated at 4°C for 30 min with anti-HA-Alexa 488 (Life Technologies) diluted in DMEM (1:300) followed by brief washing in cold dPBS and incubation in serum-free DMEM for various periods of time at 37°C. Cells were then washed in cold dPBS and fixed with 4% PFA.

### Immunocytochemistry and microscopy

For immunocytochemistry cells were plated on coverslips 24 hours before the experiment. The cells were fixed for 10 min in 4% cold PFA, washed in dPBS and permabilised in 0.3% Triton X-100 (in dPBS) for 10 min at room temperature. After washing and incubation in blocking buffer (0.1% Tween-20, 10% NGS (normal goat serum), 0.3 M Gycine in dPBS) for at least 30 min, cells were incubated with primary antibodies in staining buffer (0.1% Tween-20, 1.5% NGS in dPBS) overnight at 4°C. The following day, the cells were washed in dPBS and incubated with secondary antibodies (1:500 diluted in staining buffer) for an hour at room temperature. Subsequently, after another washing step the nuclei were stained with DAPI (3μg/mL) and mounted with VECTASHIELD (Vector Laboratories) on microscope slides. Next, the cells were analysed using an Apotome microscope (Axio Imager Z1m, Zeiss). Representative images were processed and brightness of images was uniformly adjusted to enhance contrast using Adobe Photoshop. For colocalisation studies the AxioVision Colocalization Module was used. The following antibodies were used in a 1:200–500 dilution: Calnexin (Cell Signaling Technology, 2433), CC2D1A/mLGD2 (this study), CHMP4B (Santa Cruz, C12), LAMP1 (DSHB, 1D4B), RAB5 (abcam, ab18211), RAB7 (abcam, ab50533), SYNTAXIN 6 (Cell Signaling Technology, 2869), γ-Tubulin (Sigma, T5326). CC2D1A antibodies that gave unspecific signals in *Cc2d1a* deficient MEFs were the following: Bethyl Lab (CC2D1A, A300-285A), Santa Cruz Biotechnology (Freud-1 (K19), sc-79482), Abnova (CC2D1A, H00054862-B01P) and a customized rabbit polyclonal peptide antibody directed against residues 577–592 (GenScript, [10])

### Proximity ligation assay (PLA)

Cells were seeded on coverslips in 24 well dishes and cultivated overnight. Cells were then washed in dPBS and fixed for 10 min in cold 4% PFA. After washing and permeabilisation for 10 min in 0.3% Triton X-100 cells were incubated in blocking buffer (10% NGS, 0.1% Tween-20, 0.3 M Glycine in dPBS) for at least 1 hour at room temperature. Cells were then incubated with primary antibodies (CC2D1A/mLGD2 (this study) or PGK1 (Gene Tex, GTX107614) and CHMP4B (Santa Cruz, C12)) in staining solution (1.5% NGS, 0.1% Tween-20 in dPBS) overnight at 4°C, followed by 1 hour incubation with PLA probes. The PLA Probe for the CC2D1A/mLGD2 antibody was generated using the Duolink In Situ Probemaker Minus Kit (Olink Bioscience) and anti-guinea pig IgG (Jackson Immuno Research) according to manufacturers`instructions and for the CHMP4B antibody the Duolink In Situ PLA probe anti-rabbit was used. The next steps were performed according to the Duolink In Situ protocol provided by Olink Bioscience. For detection the Duolink In Situ detection reagents red were used. Statistical analysis was performed using Student`s t-test, unpaired, two-tailed.

### EM analysis

Cells were grown to confluency in 96 well dishes on Aclar films, fixed for 30 min in fixing solution (2.0% glutaraldehyde, 0.2% saturated picric acid in 0.1 M cacodylate buffer) washed in 0.1 M cacodylate buffer and postfixed in 1% osmium tetroxide in 0.1 M cacodylate buffer for 30 min. The specimens were dehydrated in EtOH and embedded in Epon using ethanol as an intermediate solvent. Thin sections were contrasted for 5 min in 2% uranyl acetate and 4 min in Reynolds lead citrate and observed under an EM 902 (Zeiss) microscope at 80 KV. For the quantification of the perimeter of endosomal and lysosomal areas, images of whole cells were acquired, as well as higher magnifications of all parts which contained endosomal compartments. ImageJ was used to trace the membrane of endosomes and lysosomes and the perimeter was measured. At least four individual experiments were performed and at least 35 cells for each genotype were analysed. Statistical analysis was performed using Student`s t-test, unpaired, two-tailed.

### *D*. *melanogaster* genetics

The human CC2D1A and CC2D1B constructs were generated by PCR using synthesized CC2D1B (Ensembl transcript ENST00000284376, Genscript) or CC2D1A (Clone ID: 6585236, Source BioScience) as templates. Amplified sequences were cloned into plgdPattB using *Not*I and *Kpn*I or *Xho*I restriction sites [22]. Primer sequences are available upon request. All constructs were sequenced prior to injection. *D*. *melanogaster* lines: *lgd* ^d7^ FRT40A [65] and *shrub* ^4-1^ FRTG13 [27]. Antibody staining was performed according to standard protocols. Anti-wingless antibody was purchased from DSHB (4D4) and nuclei were stained with Hoechst 33258 dye. Images were obtained with an Apotome microscope (Axio Imager Z1m, Zeiss).

## Supporting Information

S1 Fig Loss of CC2D1A results in reduced levels of CC2D1B. (A) Immunoblotting of wild type, Cc2d1a^-/-^ and Cc2d1b^-/-^ MEF cells with CC2D1A and CC2D1B antibodies, respectively. Quantification of CC2D1B (B, n = 2) and CC2D1A (C, n = 4) levels normalised to α-Tubulin revealed reduced expression of CC2D1B in *Cc2d1a* ^-/-^ MEFs.
(TIF)

S2 Fig CC2D1A antibodies bind unspecific in Cc2d1a^-/-^ cells. Immunocytochemical staining on wild type and *Cc2d1a* ^-/-^ MEFs with antibodies directed against CC2D1A. All tested CC2D1A antibodies gave strong signals in wild type and in *Cc2d1a* deficient cells. Scale bars are 20 μm.
(TIF)

S3 Fig The number of goblet cells is not altered in *Cc2d1a* or *Cc2d1b* deficient intestines. Alcian blue staining was performed on intestinal sections of three animals per genotype and the number of goblet cells was counted in 35–46 250μm crypt-villus units per animal. No significant differences were found in the amount of goblet cells in intestines of *Villin-Cre* ^*tg/+*^,*Cc2d1a* ^flox/flox^ (11.91±2.20, p = 0.28) and *Cc2d1b* ^-/-^ (11.99±2.20, p = 0.41) animals compared to control animals (12.24±2.51).
(TIF)

S4 Fig CC2D1A is located in the cytosol. Immunocytochemical staining on wild type MEFs with antibodies directed against CC2D1A and the ER marker CALNEXIN (A), the Golgi marker SYNTAXIN 6 (B), the early endosomal marker RAB5 (D), the late endosomal marker RAB7 (C) and the late endosome/lysosome marker LAMP1 (E). No co-localisation between CC2D1A and the analysed markers was detected. Scale bars are 20 μm.
(TIF)

S5 Fig CC2D1A and CC2D1B are located in the cytosol and on endosomal membranes. (A) Distribution of CC2D1A, CC2D1B and CHMP4B between the cytosol (C) and membrane (M) fractions isolated from wild type, *Cc2d1a* ^*-/-*^ and *Cc2d1b* ^*-/-*^ MEFs. Cytosol and membranes from indicated cells were separated and evaluated by immunoblotting with indicated antibodies. PGK1 and LAMP1 served as control proteins for the purity of cytosol and membrane fractions, respectively. CC2D1A, CC2D1B and CHMP4B are enriched in both fractions. (B) Immunocytochemical staining was performed with indicated antibodies on wild type MEFs over-expressing either VPS4B or the dominant negative VPS4B^E235Q^. In contrast to over-expression of VPS4B, only over-expression of VPS4B^E235Q^ led to the formation of enlarged endosomes. There CC2D1A, CHMP4B and FK2, a marker for ubiquitinated proteins, accumulate. (B`) Colocalisation was assessed by measuring the Pearson`s correlation coefficient (PCC) (n≥10). (C) Immunocytochemical staining was performed with indicated antibodies on *Vps4a* ^+/-^;*Vps4b* ^+/-^ MEFs over-expressing either human CC2D1B-HA or GFP-humanCC2D1B. CC2D1B co-localises with LBPA and LAMP1 on enlarged endosomes. Scale bars are 20 μm.
(TIF)

S6 Fig Endogenous CHMP4B and PGK1 do not interact in wild type MEFs. PLA (Proximity Ligation Assay) was performed on wild type MEFs. Positive signals were abundant only in the positive control (CHMP4B and CC2D1A, 5.61±2.39 dots/cell) while CHMP4B and PGK1 display very few signals (0.64±1.01). Data are mean ± SD values from more than 80 cells/experiment (** p < 0.001). Scale bars are 20 μm.
(TIF)

S7 Fig Loss of *Cc2d1a* function results in an enlargement of the endo/lysosomal compartment. Examples of different stages of endolysosomal and auto(phago)lysosomal structures in wild type cells that were analysed for size distribution (A-D). Shown are early MVBs (C yellow arrows) and endolysosomal stages, where ILVs (D, yellow arrows) or only membranous structures (A, yellow arrows) could be observed. Late lysosomal/auto(phago)lysosomal structures (B, yellow arrow) were also measured. The perimeter of endosomal and lysosomal vesicles of wild type, *Cc2d1a* ^-/-^, *Cc2d1b* ^-/-^ and Vps4a^+/-^;Vps4b^+/-^ MEFs was measured and allocated to different size classes. *Cc2d1a* ^-/-^ and Vps4a^+/-^;Vps4b^+/-^ cells contained classes of large endo/lysosomes that were not present in *Cc2d1b* ^-/-^ or wild type cells. At least four individual experiments were performed and at least 35 cells for each genotype were analysed. Scale bar is 0.5μm (A-D).
(TIF)

S1 Table *Nestin-Cre* ^+/-^;*Cc2d1a* ^flox/+^ x *Cc2d1a* ^flox/flox^ breedings. Genotype distribution of offspring at weaning age (N = 222, χ^2^ = 78.2, p = 7x10^-8^).
(TIF)

S2 Table *Nestin-Cre* ^+/-^;*Cc2d1a* ^flox/+^ x *Cc2d1a* ^flox/flox^ breedings. Genotype distribution of embryos between stages E13.5 and E18.5 (N = 33, χ^2^ = 4.46, p = 0.22).
(TIF)

S3 Table *Nestin-Cre* ^+/-^;*Cc2d1a* ^flox/+^ x *Cc2d1a* ^flox/flox^ breedings. Genotype distribution of offspring at birth (N = 38, χ^2^ = 1.37, p = 0.71) and number of dead animals (†).
(TIF)

S4 Table *Cc2d1a* ^+/-^ *x Cc2d1a* ^+/-^ breedings. Genotype distribution of offspring at birth (N = 46, χ^2^ = 2.18, p = 0.35) and number of dead animals (†).
(TIF)

S5 Table *Cc2d1a* ^+/-^ x *Cc2d1a* ^+/-^ breedings (C57BL/6J). Genotype distribution of offspring at birth (N = 21, χ^2^ = 2.05, p = 0.36) and number of dead animals (†).
(TIF)
