# Supplementary figures and images for: The Mammalian Orthologs of Drosophila Lgd, CC2D1A and CC2D1B, Function in the Endocytic Pathway, but Their Individual Loss of Function Does Not Affect Notch Signalling
Source: PLoS Genet. 2015 Dec 31;11(12):e1005749. doi: 10.1371/journal.pgen.1005749 (PMC4697852; doi:10.1371/journal.pgen.1005749)

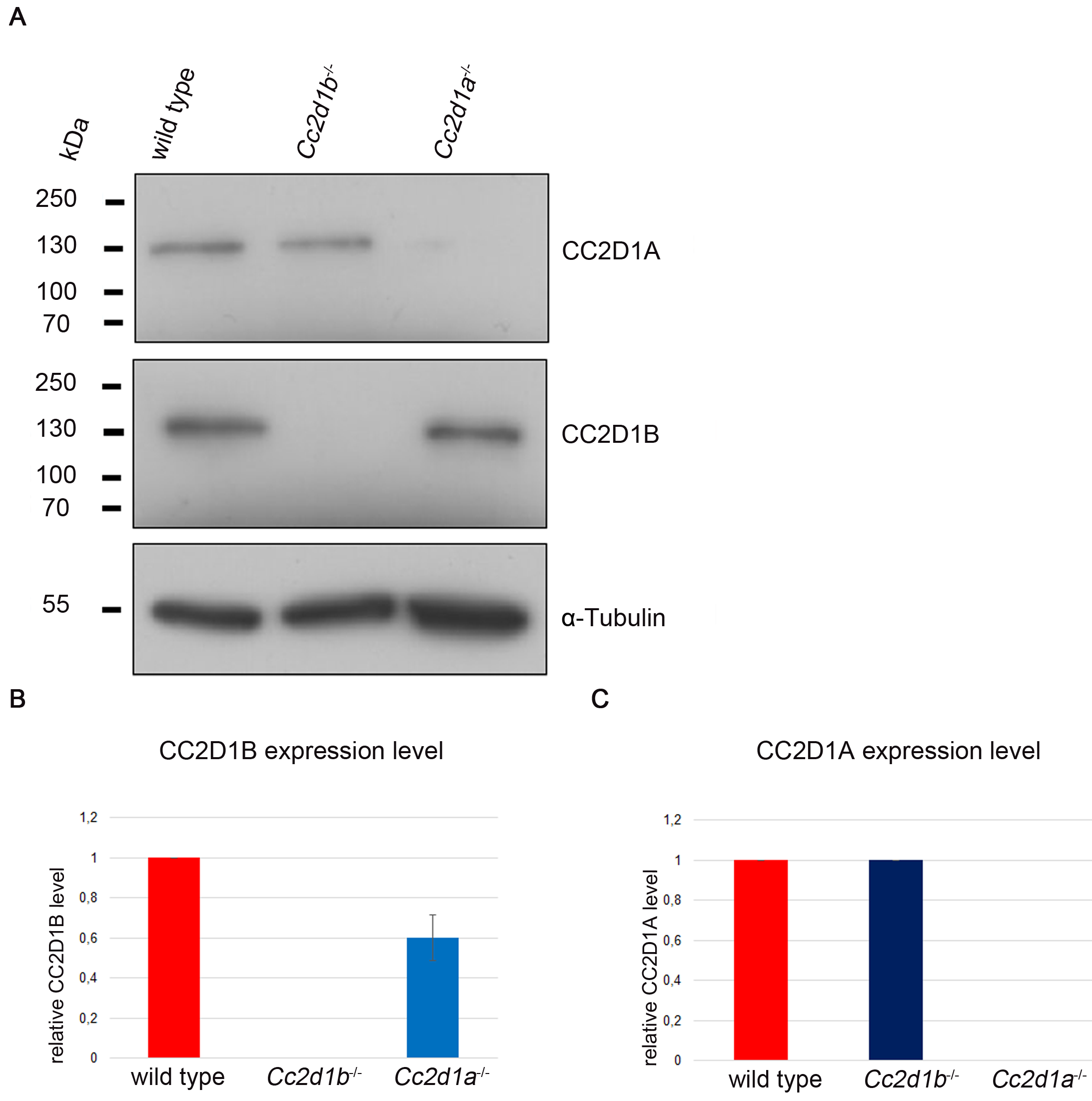

Supplement: S1 Fig — (A) Immunoblotting of wild type, Cc2d1a-/- and Cc2d1b-/- MEF cells with CC2D1A and CC2D1B antibodies, respectively. Quantification of CC2D1B (B, n = 2) and CC2D1A (C, n = 4) levels normalised to α-Tubulin revealed reduced expression of CC2D1B in Cc2d1a -/- MEFs. (TIF) [file pgen.1005749.s001.tif]

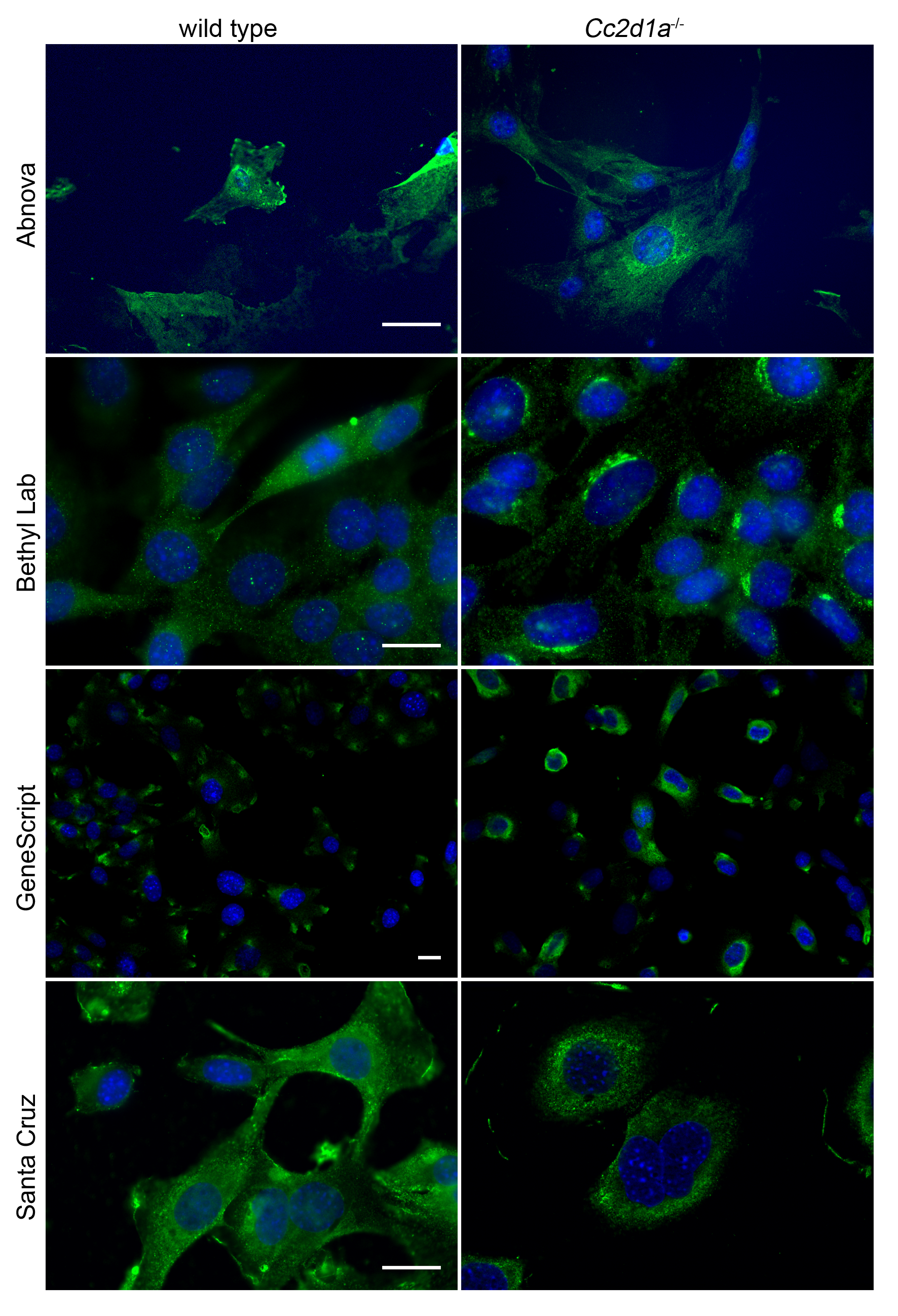

Supplement: S2 Fig — Immunocytochemical staining on wild type and Cc2d1a -/- MEFs with antibodies directed against CC2D1A. All tested CC2D1A antibodies gave strong signals in wild type and in Cc2d1a deficient cells. Scale bars are 20 μm. (TIF) [file pgen.1005749.s002.tif]

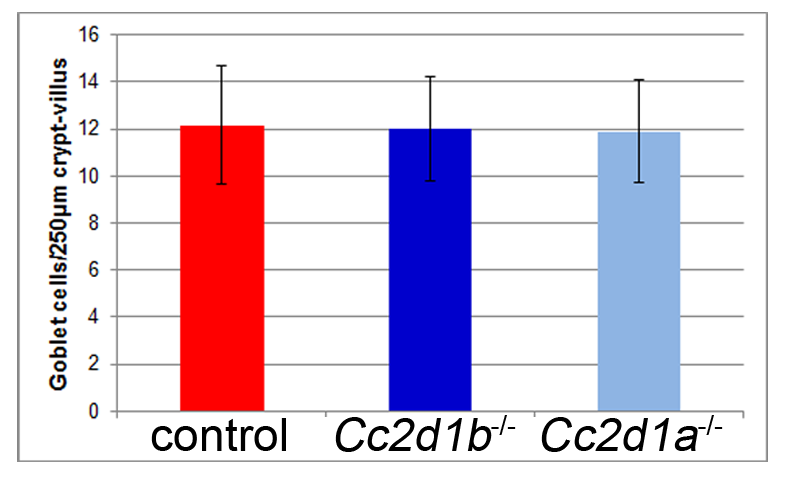

Supplement: S3 Fig — Alcian blue staining was performed on intestinal sections of three animals per genotype and the number of goblet cells was counted in 35–46 250μm crypt-villus units per animal. No significant differences were found in the amount of goblet cells in intestines of Villin-Cre tg/+,Cc2d1a flox/flox (11.91±2.20, p = 0.28) and Cc2d1b -/- (11.99±2.20, p = 0.41) animals compared to control animals (12.24±2.51). (TIF) [file pgen.1005749.s003.tif]

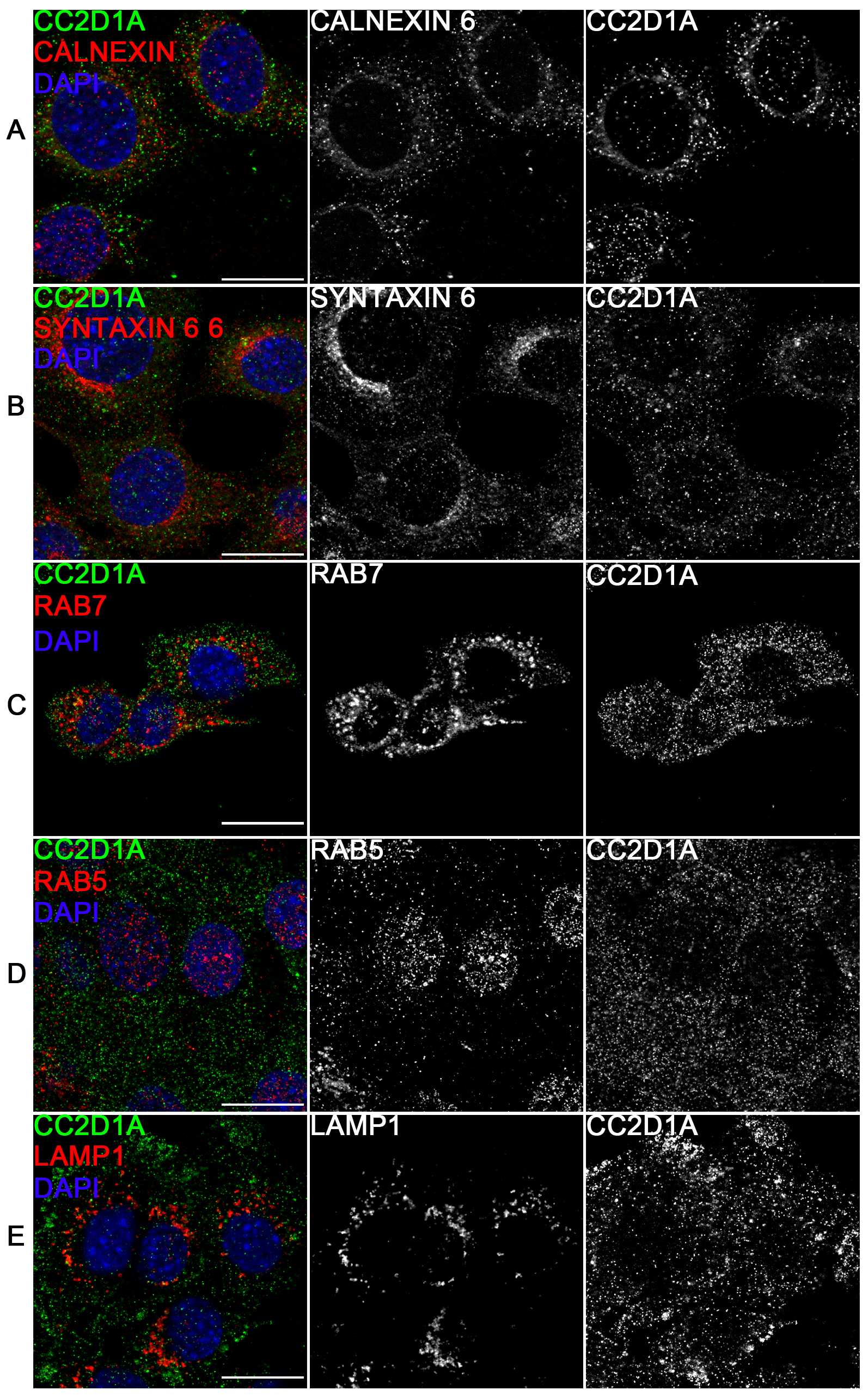

Supplement: S4 Fig — Immunocytochemical staining on wild type MEFs with antibodies directed against CC2D1A and the ER marker CALNEXIN (A), the Golgi marker SYNTAXIN 6 (B), the early endosomal marker RAB5 (D), the late endosomal marker RAB7 (C) and the late endosome/lysosome marker LAMP1 (E). No co-localisation between CC2D1A and the analysed markers was detected. Scale bars are 20 μm. (TIF) [file pgen.1005749.s004.tif]

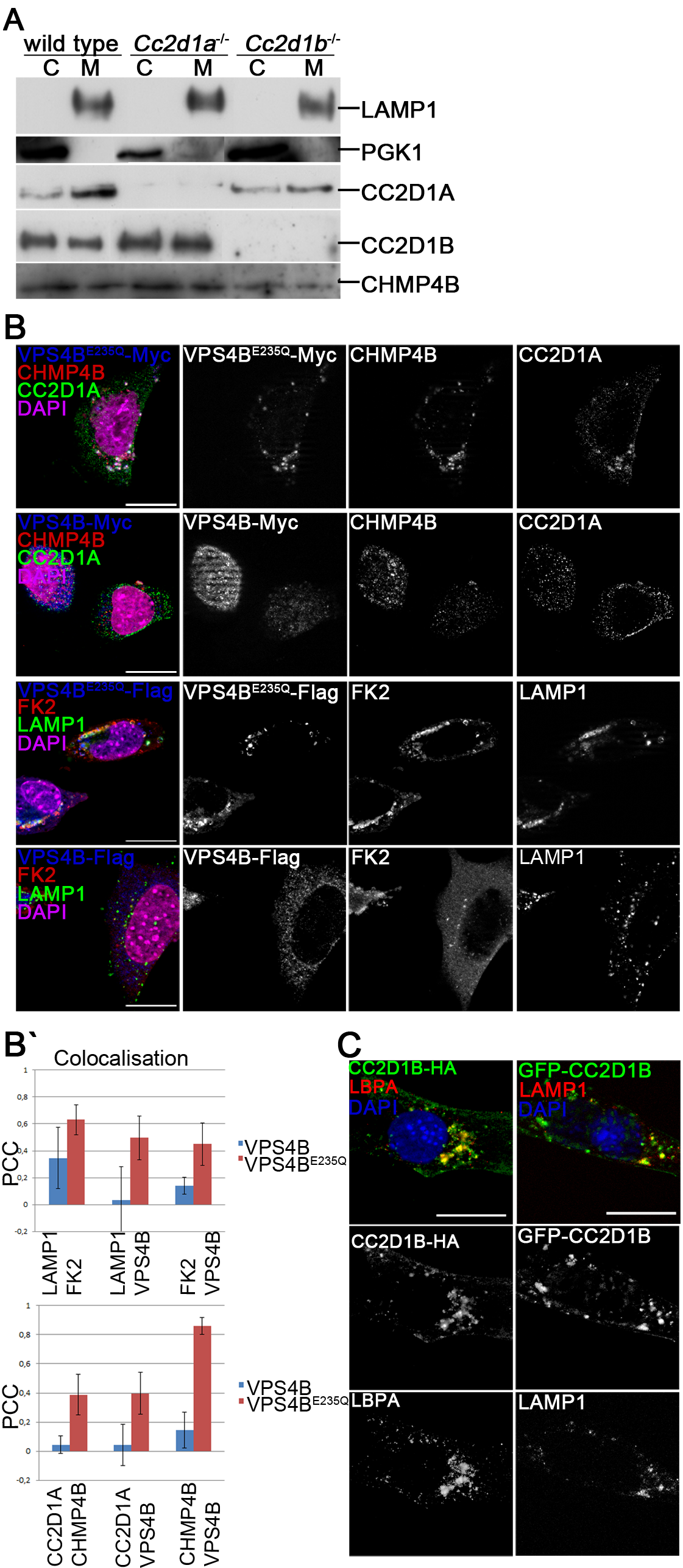

Supplement: S5 Fig — (A) Distribution of CC2D1A, CC2D1B and CHMP4B between the cytosol (C) and membrane (M) fractions isolated from wild type, Cc2d1a -/- and Cc2d1b -/- MEFs. Cytosol and membranes from indicated cells were separated and evaluated by immunoblotting with indicated antibodies. PGK1 and LAMP1 served as control proteins for the purity of cytosol and membrane fractions, respectively. CC2D1A, CC2D1B and CHMP4B are enriched in both fractions. (B) Immunocytochemical staining was performed with indicated antibodies on wild type MEFs over-expressing either VPS4B or the dominant negative VPS4BE235Q. In contrast to over-expression of VPS4B, only over-expression of VPS4BE235Q led to the formation of enlarged endosomes. There CC2D1A, CHMP4B and FK2, a marker for ubiquitinated proteins, accumulate. (B`) Colocalisation was assessed by measuring the Pearson`s correlation coefficient (PCC) (n≥10). (C) Immunocytochemical staining was performed with indicated antibodies on Vps4a +/-;Vps4b +/- MEFs over-expressing either human CC2D1B-HA or GFP-humanCC2D1B. CC2D1B co-localises with LBPA and LAMP1 on enlarged endosomes. Scale bars are 20 μm. (TIF) [file pgen.1005749.s005.tif]

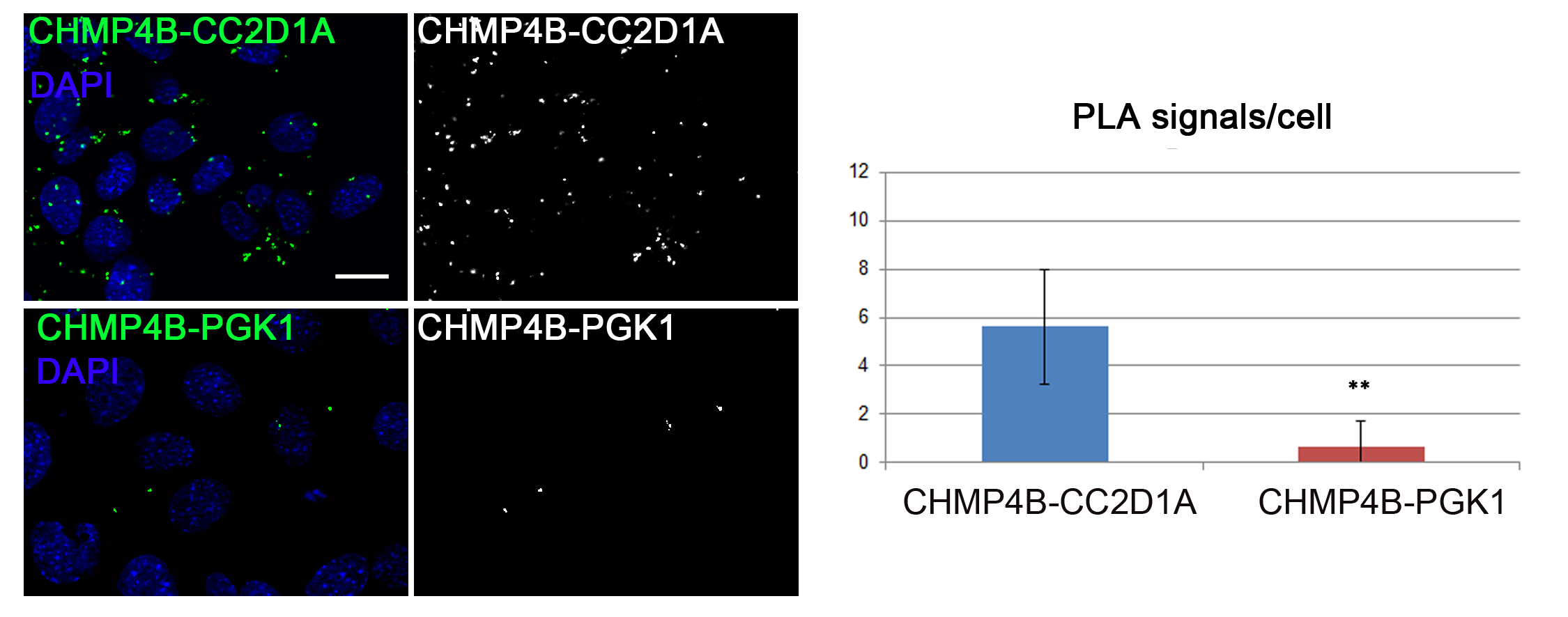

Supplement: S6 Fig — PLA (Proximity Ligation Assay) was performed on wild type MEFs. Positive signals were abundant only in the positive control (CHMP4B and CC2D1A, 5.61±2.39 dots/cell) while CHMP4B and PGK1 display very few signals (0.64±1.01). Data are mean ± SD values from more than 80 cells/experiment (** p < 0.001). Scale bars are 20 μm. (TIF) [file pgen.1005749.s006.tif]

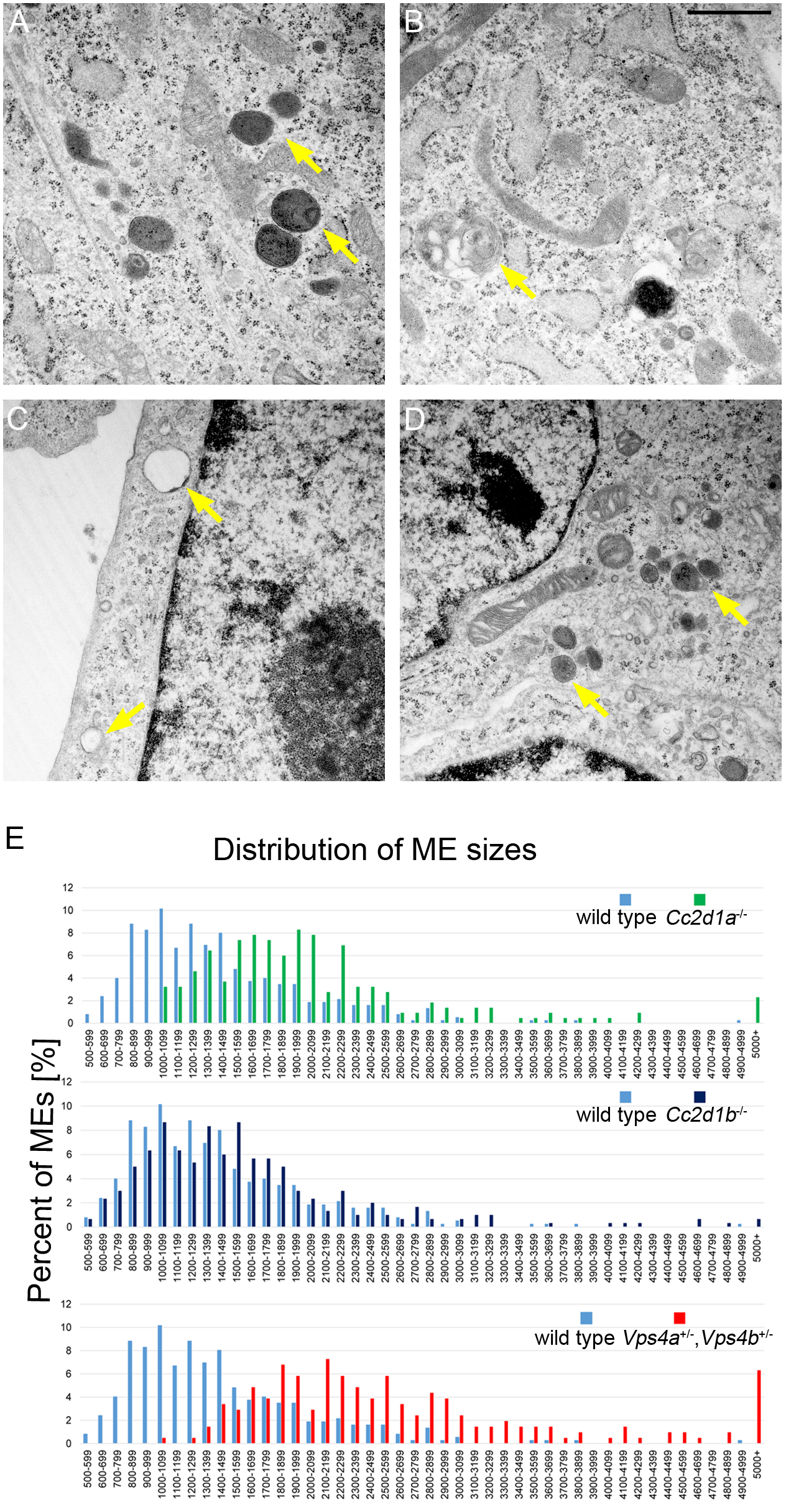

Supplement: S7 Fig — Examples of different stages of endolysosomal and auto(phago)lysosomal structures in wild type cells that were analysed for size distribution (A-D). Shown are early MVBs (C yellow arrows) and endolysosomal stages, where ILVs (D, yellow arrows) or only membranous structures (A, yellow arrows) could be observed. Late lysosomal/auto(phago)lysosomal structures (B, yellow arrow) were also measured. The perimeter of endosomal and lysosomal vesicles of wild type, Cc2d1a -/-, Cc2d1b -/- and Vps4a+/-;Vps4b+/- MEFs was measured and allocated to different size classes. Cc2d1a -/- and Vps4a+/-;Vps4b+/- cells contained classes of large endo/lysosomes that were not present in Cc2d1b -/- or wild type cells. At least four individual experiments were performed and at least 35 cells for each genotype were analysed. Scale bar is 0.5μm (A-D). (TIF) [file pgen.1005749.s007.tif]

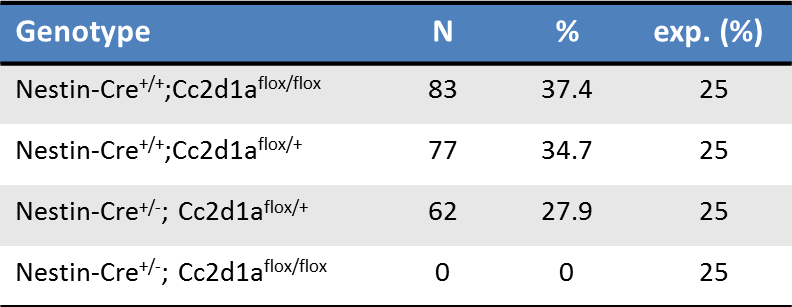

Supplement: S1 Table — Genotype distribution of offspring at weaning age (N = 222, χ2 = 78.2, p = 7x10^-8^). (TIF) [file pgen.1005749.s008.tif]

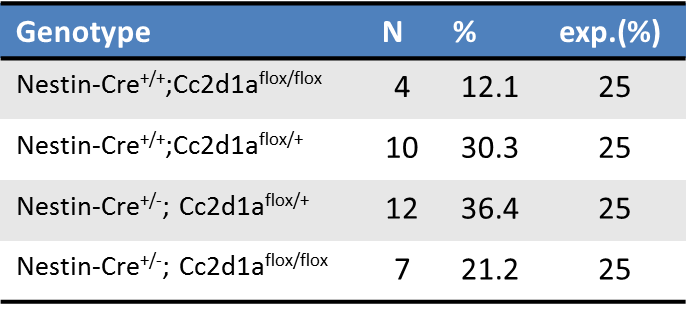

Supplement: S2 Table — Genotype distribution of embryos between stages E13.5 and E18.5 (N = 33, χ2 = 4.46, p = 0.22). (TIF) [file pgen.1005749.s009.tif]

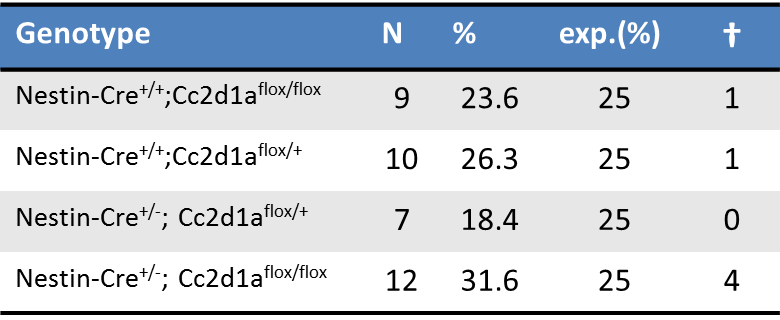

Supplement: S3 Table — Genotype distribution of offspring at birth (N = 38, χ2 = 1.37, p = 0.71) and number of dead animals (†). (TIF) [file pgen.1005749.s010.tif]

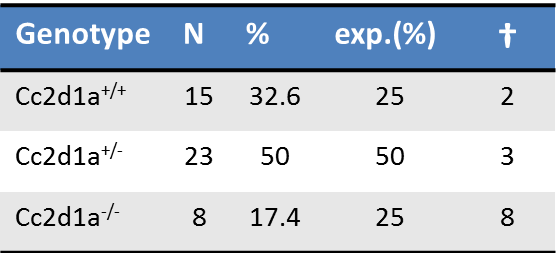

Supplement: S4 Table — Genotype distribution of offspring at birth (N = 46, χ2 = 2.18, p = 0.35) and number of dead animals (†). (TIF) [file pgen.1005749.s011.tif]

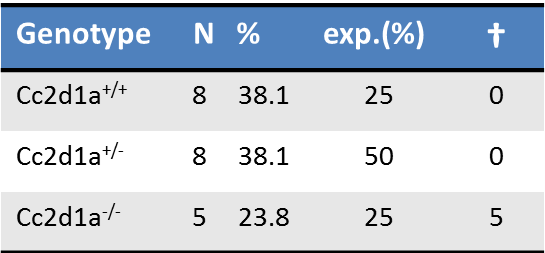

Supplement: S5 Table — Genotype distribution of offspring at birth (N = 21, χ2 = 2.05, p = 0.36) and number of dead animals (†). (TIF) [file pgen.1005749.s012.tif]
